# Efficient Lignin Dissolution and Enzymatic Depolymerization in Ethylene Glycol-Based Deep Eutectic Solvent Mixtures: Integrated Experimental and Molecular Dynamics Study

**DOI:** 10.1021/acs.jpcb.5c07451

**Published:** 2026-02-11

**Authors:** Julian Schilke, Miriam Sprick, Eduardo Schneider, Josué Arturo Ledezma Fierro, Alexandre Pradal, Gabriele Raabe, Anett Schallmey

**Affiliations:** † 26527 Institute for Biochemistry, Biotechnology and Bioinformatics, Technische Universität Braunschweig, Spielmannstraße 7, Braunschweig 38106, Germany; ‡ Institute of Thermodynamics, Technische Universität Braunschweig, Hans-Sommer-Straße 5, Braunschweig 38106, Germany; § Center of Pharmaceutical Engineering, Technische Universität Braunschweig, Franz-Liszt-Straße 35a, Braunschweig 38106, Germany; ∥ Institut Parisien de Chimie Moléculaire, 517735 Sorbonne Université, 4 Place Jussieu, Paris 75005, France

## Abstract

Lignin is a promising starting material for the future provision of aromatic platform chemicals. Its low solubility in aqueous media, however, hampers its efficient depolymerization using enzymatic approaches. Thus, the addition of cosolvents is often required to improve the lignin solubility. Herein, we demonstrate that mixtures of ethylene glycol/choline chloride (EG:ChCl)-based deep eutectic solvents (DES) and glycine buffer represent powerful solvent systems for lignin dissolution in β-etherase-catalyzed lignin depolymerization reactions. They enabled the use of high lignin concentrations (up to 30 g L^–1^ at 60% v/v DES) without abolishing enzyme activity. While EG:ChCl 2:1 proved superior in solubilizing lignin, the highest volumetric yield of the desired monoaromatic depolymerization products guaiacylhydroxypropanone and syringylhydroxypropanone was achieved using 20 g L^–1^ lignin and 50% v/v EG:ChCl 4:1. Moreover, detailed MD analyses on the interactions of the lignin model compound guaiacylglycerol-β-guaiacyl ether (GGE) and the solvent mixture, including also glycine as a buffer component, highlight the importance of EG and glycine for lignin solubility, while the ratio of EG and ChCl in the DES impacts the solvation structure and dynamics of GGE in the solvent mixture. Thus, our comprehensive study offers insights into the molecular determinants that affect lignin solubility and its enzymatic depolymerization.

## Graphical abstract

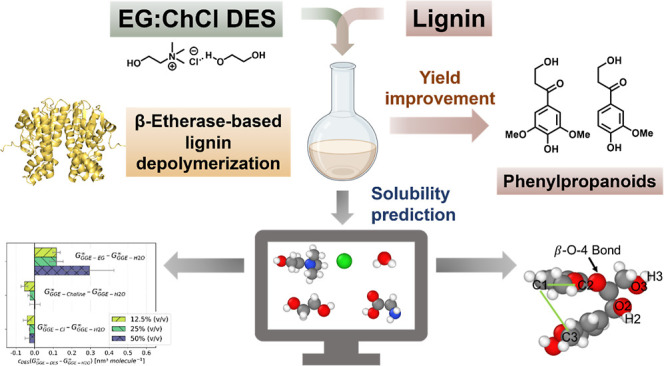

## Introduction

In the face of growing global demand for renewable resources, the conversion of biomass into sustainable fuels and chemicals plays a critical role in the transition to an economy with a low CO_2_ footprint. Lignin, the most abundant renewable aromatic polymer on Earth,^1^ holds significant promise in this context. It is cost-effective, environmentally benign,^2^ and a potential substitute for petroleum-derived aromatic compounds in the chemical industry.^3^

Lignin is composed mainly of G, S, and H phenylpropanoid units that are derived from the three monolignols *p*-coumaryl alcohol (H), coniferyl alcohol (G), and sinapyl alcohol (S), which are coupled via an oxidative radical mechanism during lignin biosynthesis in the plant cell wall. This results in the formation of various C–O and C–C bonds, with the β-*O*-4 aryl ether bond representing the most abundant linkage type in lignin, accounting for 45–60% of total linkages present.^4–6^ Lignin can be isolated from lignocellulosic biomass using either physical, chemical, physicochemical, or biological methods.^7–9^ Among these, the most common chemical lignin extraction method is the Kraft process, which is used in the pulp and paper industry. Like many others, it uses harsh conditions to separate lignin from the carbohydrate fractions (i.e., cellulose and hemicellulose) of lignocellulose, resulting in a loss of the weaker C–O bonds and a marked change in lignin structure.^8,10,11^ To conserve the native linkages within lignin for later valorization, milder processes are available, such as chemical extraction via alkaline, acidic, or organosolv treatment.^9^ In the commonly used organosolv process, aqueous organic solvents such as ethanol, ethylene glycol, or 2-methyltetrahydrofuran are used in combination with an acid or base catalyst.^12–14^ This results in a lignin of high purity and native-like structure, making it attractive for later valorization.^15^ Additionally, lignin isolation methods involving ionic liquids or deep eutectic solvents (DES) have been developed,^16–18^ as those solvents are very powerful in solubilizing lignin.

Subsequently, either chemical or biocatalytic lignin depolymerization strategies can be employed in order to obtain valuable monoaromatic products from lignin. Available chemical depolymerization strategies (e.g., base- or acid-mediated as well as oxidative and reductive approaches) commonly target different linkage types within the lignin and are often performed under mild conditions to yield a range of degradation products.^8^ Alternatively, biocatalytic approaches for lignin depolymerization make use of the enzymatic repertoire of lignin-degrading fungi and bacteria.^19^ This includes extracellular laccases and peroxidases (i.e., lignin peroxidases, manganese peroxidases, versatile peroxidases, and dye-decolorizing peroxidases),^20–22^ which use either oxygen or H_2_O_2_ as an oxidizing agent for lignin degradation, often in combination with mediators. As these oxidative enzymes rely on radical-based mechanisms, lignin depolymerization happens in an unselective fashion, resulting in a broad spectrum of degradation products. In contrast, glutathione-dependent β-etherases selectively cleave the β-*O*-4 linkages in lignin via a nonradical mechanism.^4,20,23^ These enzymes are part of a metabolic pathway, mostly found in bacteria,^24–27^ which starts with the oxidation of the Cα hydroxyl groups in lignin catalyzed by enantioselective Cα-dehydrogenases. In the next step, enantioselective β-etherases cleave the β-*O*-4 aryl ether linkages with the help of glutathione (GSH) and the formation of a glutathione adduct. The latter is further converted by GSH-dependent glutathione lyases, which results in thioether cleavage and the release of oxidized glutathione (GSSG). By combining these enzymes in an in vitro cascade for the depolymerization of lignin (Figure 1), guaiacylhydroxypropanone (GHP) and syringylhydroxypropanone (SHP) are selectively obtained as the major degradation products when starting from wood-derived lignins.^15,28–32^ Hence, this enzymatic approach to lignin valorization is particularly promising, but it has been demonstrated for only low lignin concentrations up to 2 g L^–1^ so far, while higher substrate concentrations would be required to achieve economically feasible product titers. Since enzymatic reactions take place in a liquid phase, usually water-based, the choice of solvent system for substrate dissolution is crucial. Lignin is inherently difficult to solubilize in aqueous media,^33^ but DES have emerged as a powerful and versatile class of cosolvents capable of overcoming this limitation by acting as hydrotropes in lignin dissolution.^34–37^ For example, the ethylene glycol/choline chloride (2:1) DES, also called ethaline, has been reported to solubilize Kraft lignin up to 25% (w/w), whereas the DES consisting of propionic acid/urea (2:1) enables lignin concentrations of up to 80% (w/w).^35,37–39^ At the same time, DES are usually more biocompatible and less toxic than ionic liquids.^40^ Moreover, DES have been applied in various enzymatic reactions and their impact on enzyme stability and activity has been studied,^41^ but they have not been investigated as suitable cosolvents in combination with the enzyme cascade (Figure 1) for lignin depolymerization so far.

**Figure 1:**
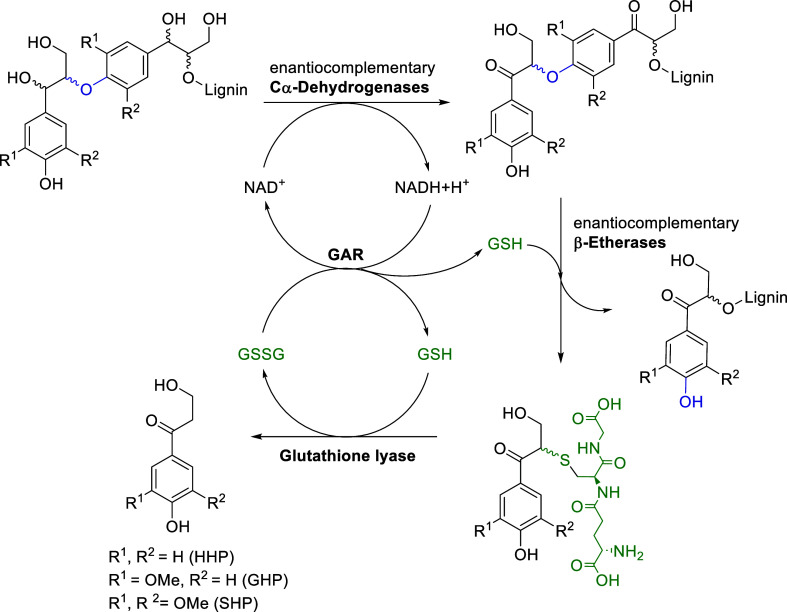
Lignin depolymerization by an enzymatic cascade composed of enantiocomplementary Cα dehydrogenases, enantiocomplementary β-etherases, and a glutathione lyase, including also cofactor recycling via a glutathione amide reductase (GAR).^29^

The main challenge associated with DESs lies in their high tunability.^42^ These solvents are generally composed of two individual components, a hydrogen bond donor (HBD) and a hydrogen bond acceptor (HBA), that can be combined in a variety of molar ratios, resulting in a broad spectrum of physicochemical properties.^43–45^ This high degree of flexibility, while advantageous for tailoring specific applications, also increases the complexity of systematic optimization. Moreover, the addition of water or salts has been shown to significantly influence the solubilization performance of lignin, further expanding the already vast design space of potential solvent systems. This complexity is further amplified by the structural heterogeneity of lignin.^46^ Rather than being a single, well-defined molecule, lignin is a highly variable polymer with a molecular structure strongly dependent on its biological source. As a result, the chemical space in which enzymatic depolymerization of lignin may occur is both broad and variable.

Due to the difficulties in capturing the complex and heterogeneous nature of lignin, theoretical studies on lignin dissolution employ lignin model compounds with simplified structures that serve as representations of native lignin. Several COSMO-RS-based screening tools to identify suitable molecular solvents and to predict lignin solubility are available in literature.^46–48^ Motivated by the need to eliminate the time-consuming QM calculations in COSMO-RS, solvent screening tool sets combining COSMO-RS with machine learning models were also recently proposed.^33,49^ Although these tools are very helpful for a pure solvent screening for lignin, they are not suited to identifying amphiphilic cosolvents that act as hydrotropes to increase the solubility of lignin in water. This is because COSMO-RS does not allow us to resolve the actual solvation structures in such complex solvent systems to capture the mechanistic details of dissolution.

Another method to describe solvent properties in screening approaches is Kamlet–Taft (K–T) solvatochromic parameters (π*, α, and β) that characterize solvents by their polarity (π*), hydrogen bonding (HB) donating capacity (α), and HB accepting ability (β).^50–52^ Thereby K–T parameters are used in linear solvent energy relationships to allow for the prediction of solute solubilities and to identify suitable K–T parameter ranges for potential solvents. Zhang et al.^53^ experimentally determined the K–T parameters for 56 DES and analyzed their relationship with the solubility of the main components of lignocellulose including lignin. Sumer and van Lehn^54^ used a regression model trained on COSMO-RS simulations to predict lignin pentamer activity coefficients and combined it with K–T parameters to characterize potential solvents. With this, they were able to analyze structure–solubility relationships and to explore solubility trends of this lignin model compound in a variety of organic solvents. However, the applicability of K–T parameters to solvent mixtures is constrained by the current lack of mixture-specific K–T parameters. Although there are some approaches available on modeling and predicting K–T parameters of mixtures,^55,56^ they fail to describe the sigmoid curves observed in mixtures of water and hydrotropes.^56^ These sigmoid trends can be attributed to microheterogeneities, i.e., local concentration variations referred to as preferential solvation. A fundamental molecular-based understanding of the solute’s relative affinity for each solvent compound and the resulting microheterogeneities in the solvent mixtures is though, essential to control the dissolution of the solute.^57^

Molecular dynamics simulations (MD) allow us to gain a detailed insight into molecular mechanisms of preferential solvation in water + hydrotrope solvent mixtures by analyzing the underlying molecular interaction patterns and resulting solvation structures.^58^ Thus, they can make an important contribution to bridging the knowledge gap between these molecular-level interaction characteristics and macroscopic solubility, which is essential for rational hydrotrope selection in lignin processing. There are already MD studies available on characterizing lignin-DES interactions and providing insight into HB preferences.^59,60^ However, to the best of our knowledge, no MD simulation studies have yet explored in detail the molecular mechanism behind the hydrotropic behavior of DES in enhancing lignin’s water solubility. Furthermore, the effect of buffer compounds on lignin dissolution has not yet been investigated by MD studies.

To identify a suitable solvent system that would enable the use of higher lignin concentrations in enzymatic lignin depolymerization applying the enzyme cascade shown in Figure 1, we herein investigated different solvent mixtures composed of DES as a hydrotrope and glycine buffer as an aqueous compound. Among the tested DES, ethylene glycol/choline chloride (EG:ChCl) turned out to be the best in terms of lignin dissolution and enzyme cascade performance, enabling effective depolymerization reactions with lignin concentrations of up to 30 g L^–1^. Moreover, to investigate the molecular mechanism underlying the EG:ChCl-induced enhanced lignin solubility in glycine buffer, we present a detailed MD study on the interactions of GGE as a lignin model compound and the solvent mixture. We provide a detailed analysis of the structural features for the DES + (water + glycine) solvent mixtures and of the GGE’s relative affinity for the different solvent compounds. With this, we offer insights into the molecular determinants that affect both the lignin solubility and its enzymatic depolymerization.

## Methods

### Chemicals

1-(4-Hydroxy-3-methoxyphenyl)-2-(2-methoxyphenoxy)­propane-1,3-diol (GGE) was purchased from TCI (Portland, USA). SHP and GHP were provided by Dr. Alexandre Pradal from the Institut Parisien de Chimie Moléculaire of the Sorbonne University (Paris, France).^61^ The Organosolv lignin K053 derived from beech wood was provided by Dr. Ing. Marlen Verges from the Fraunhofer CBP (Leuna, Germany).

Seven DES^37,62–66^ used in this study were produced by mixing the two components overnight in their respective molar ratios (Table 1) under stirring at 70 °C. All seven DES were liquid at room temperature; however, the DES Res:ChCl (1:1) and Gu:ChCl (2:1) exhibited highly viscous behavior. For the latter application in lignin depolymerization, these two DES were diluted with H_2_O to obtain less viscous mixtures, resulting in 80% (v/v) DES systems.

**Table 1:** DES Used in This Study

HBD	HBA	HBD/HBA ratio (molar)	abbreviation
ethylene glycol	choline chloride	2:1	EG:ChCl 2:1
ethylene glycol	choline chloride	3:1	EG:ChCl 3:1
ethylene glycol	choline chloride	4:1	EG:ChCl 4:1
ethylene glycol	betaine	3:1	EG:Bet 3:1
ethylene glycol	betaine	4:1	EG:Bet 4:1
guaiacol	choline chloride	2:1	Gu:ChCl 2:1
resorcinol	choline chloride	1:1	Res:ChCl 1:1

### Synthetic Genes

Synthetic genes encoding the Cα dehydrogenases LigN (WP_014077904) and LigD (WP_014075190) from *Sphingobium* sp. strain SYK-6, the β-etherase LigF-261 (WP_104831261) from *Marinicaulis flavus*, the glutathione lyase NaGST_Nu_ (WP_011446237) from *Novosphingobium aromaticivorans*, and the glutathione amide reductase TtGAR (WP_153973747) from *Thermochromatium tepidum* were purchased from Twist Bioscience (San Francisco, USA) with codon optimization for expression in *Escherichia coli* and already cloned into the expression vector pET28a­(+) from Merck (Darmstadt, Germany). The gene encoding β-etherase LigE-NA (WP_011446047) from *N. aromaticivorans* DSM 12444 had previously been cloned into the same vector.^25^ Expression of respective genes in *E. coli* BL21 (DE3) (NEB, Frankfurt, Germany) resulted in either an N-terminal (LigN, LigD, LigF-261, LigE-NA, and *Na*GST_Nu_) or C-terminal His6-tag (*Tt*GAR).

### Protein Production and Purification

Enzyme production was performed by inoculating 450 mL of TB media (12 g L^–1^ peptone, 24 g L^–1^ yeast extract, 0.4% (v/v) glycerol, 12.5 g L^–1^ K_2_HPO_4_, 2.3 g L^–1^ KH_2_PO_4_) containing 50 mg L^–1^ kanamycin and 0.1 mM IPTG with 50 mL of a respective overnight culture and incubation for 28 h at 22 °C with shaking at 200 rpm. The cells were harvested by centrifugation (20 min, 3488 g, 4 °C) and resuspended in 30 mL lysis buffer (20 mM K_2_HPO_4_, 500 mM NaCl, 20 mM MgSO_4_, 20 mM imidazole, pH 7.4) containing 30 mg of lysozyme, one tablet of protease inhibitor (Pierce Protease Inhibitor Mini Tablet, Thermo Fisher Scientific) and 0.1 mg mL^–1^ DNaseI (NEB). In case of *Tt*GAR, 10% (v/v) glycerol was used instead of 500 mM NaCl in the lysis buffer. The cells were disrupted via sonication (65% amplitude, cycles of 10 s pulse and 20 s pause over 7 min) on ice, and the cell debris was removed by centrifugation at 18,000*g* for 45 min at 4 °C. The supernatant was then filtered through a 0.45 μm pore-size filter to obtain the cell-free extract. All enzymes were purified via Ni-NTA affinity chromatography on an Äkta-Start system using a 5 mL HisTrap FF-column purchased from GE Healthcare Life Science (Solingen, Germany). The column was loaded with the cell-free extract at a flow rate of 2 mL min^–1^, then the column was washed with 10 column volumes (CV) buffer A (20 mM K_2_HPO_4_, 500 mM NaCl, 20 mM imidazole, pH 7.4). The program to elute the enzymes used a flow rate of 5 mL min^–1^ and a gradient of 0–100% buffer B (20 mM K_2_HPO_4_, 500 mM NaCl, 500 mM imidazole, pH 7.4) in buffer A over 30 CV. In the case of *Tt*GAR, 10% (v/v) glycerol was used instead of 500 mM NaCl in buffers A and B. Enzyme-containing fractions were combined and concentrated to a volume of 2 mL using a Vivaspin Turbo 15 ultrafiltration unit (Sartorius, Göttingen, Germany). Afterward, the purified enzymes were desalted using a PD-10 Sephadex G-25 M column (Cytiva, Wilmington, USA) and 20 mM Tris/SO_4_ buffer, pH 7.4, containing 20% (v/v) glycerol, before storage at −20 °C. Final enzyme concentrations were determined via absorbance at 280 nm using extinction coefficients predicted by ProtParam (SIB E*x*PAS*y* Bioinformatics Resource Portal, Lausanne, Switzerland). The purity of each purified enzyme was analyzed via SDS-PAGE (Figure S1).

### Enzymatic Lignin Depolymerization

#### Comparison of Cosolvent Systems

Seven DES, as well as dimethyl sulfoxide (DMSO), were employed to compare their impact on the yields of SHP and GHP in enzymatic lignin depolymerization. First, stock solutions of 20 g L^–1^ lignin in DES or DMSO were prepared by rapid mixing over 2 h at room temperature. Lignin depolymerization reactions were performed in 250 μL volume containing 2 g L^–1^ of lignin, 400 mM glycine/NaOH buffer, pH 9, DMSO, or DES at final concentrations of 10–60% (v/v), 5 mM NAD^+^, 8 mM GSH, and 0.25 g L^–1^ of each purified enzyme. The reactions were incubated for 1 h at 30 °C with shaking at 1000 rpm in a ThermoMixer C from Eppendorf (Hamburg, Germany). Afterward, 125 μL of brine was added, and the resulting mixture was extracted three times with 750 μL of dichloromethane. The organic layers were combined, and the organic solvent was evaporated at 60 °C. Then, the dried crude was dissolved again in 1 mL of dichloromethane for subsequent analysis via HPLC.

#### Impact of Cosolvent/DES Concentration on Lignin Depolymerization

Lignin was dissolved in DMSO to a concentration of 100 g L^–1^, while in the DES EG:ChCl 2:1, 3:1, and 4:1, lignin could only be dissolved to a concentration of 50 g L^–1^. Lignin depolymerization reactions were performed in a 250 μL volume. For reactions using DMSO as a cosolvent, the reactions contained 1–40 g L^–1^ lignin, 400 mM glycine/NaOH buffer, pH 9, 5–60% (v/v) DMSO, 5 mM NAD^+^, 8 mM GSH, and 0.25 g L^–1^ of each purified enzyme. For reactions using DES as a cosolvent, the reactions contained 1–30 g L^–1^ lignin, 400 mM glycine/NaOH buffer, pH 9, 5–60% (v/v) DES, 5 mM NAD^+^, 8 mM GSH, and 0.25 g L^–1^ of each purified enzyme. All reactions were incubated for 1 h at 30 °C with shaking at 1000 rpm in a ThermoMixer C from Eppendorf (Hamburg, Germany). Afterward, 125 μL of brine was added, and the resulting mixture was extracted three times with 750 μL dichloromethane. The organic layers were combined, and the organic solvent was evaporated at 60 °C. Then, the dried crude was dissolved again in 1 mL dichloromethane for subsequent analysis via HPLC.

### Solubility Tests of Lignin Model Compound

Solubility tests of lignin model compound GGE were performed in 2 mL Eppendorf reaction tubes as duplicates. The tubes contained 400 mM glycine/NaOH (pH 9.0) and DES (EG:ChCl 2:1 or 3:1 or 4:1) in concentrations of 12.5%, 25%, 50%, and 75% (v/v) in a total volume of 1 mL. Additionally, tubes containing only the three DES without buffer addition were prepared. The tubes were then weighed to determine the density of each DES-buffer system (Table S2). The model compound GGE was afterward added in excess to prepared tubes containing 1 mL of each DES-buffer system to ensure saturation, and the tubes were incubated overnight at room temperature with shaking at 1000 rpm in a ThermoMixer C from Eppendorf (Hamburg, Germany). The next day, the tubes were centrifuged for 5 min at 17,000*g* to remove nondissolved solids. Each 50 μL of saturated solution was transferred into HPLC vials containing 950 μL of DMSO for subsequent HPLC analysis.

### HPLC Analysis

#### Analysis of Enzymatic Lignin Depolymerization

To quantify the amount of released SHP and GHP in the enzymatic lignin depolymerization, reaction samples were analyzed via reversed-phase high pressure liquid chromatography on a Nexera XR20 HPLC system from Shimadzu (Duisburg, Germany) using a Nucleosil 100–5C18 column (250 × 4 mm) purchased from Macherey Nagel (Düren, Germany). The conditions for separation included an injection volume of 10 μL, a column temperature of 40 °C, a flow rate of 1 mL min^–1^, and a gradient elution using a mixture of acetonitrile and TFA-containing water (0.08% (v/v) TFA) as the mobile phase. The gradient program was as follows: 0–1 min 10% (v/v) acetonitrile, 1–15 min linear increase from 10% to 32% (v/v) acetonitrile, 15–17 min linear increase from 32% to 100% (v/v) acetonitrile, 17–25 min 100% (v/v) acetonitrile, and 25–26 min linear decrease from 100% (v/v) to 10% (v/v) acetonitrile with an overall measurement time of 26 min. The HPLC system was coupled to a UV detector to quantify the amount of SHP and GHP at 280 nm using respective calibration curves.

#### Analysis of Dissolved Lignin Model Compound

For quantification of lignin model compound solubility in the different DES:buffer systems, the same HPLC system and column were used as described above. The conditions for separation included an injection volume of 10 μL, a column temperature of 40 °C, a flow rate of 1 mL min^–1^, and an isocratic elution using a mixture of acetonitrile (50%), water (49.9%), and TFA (0.1%) as the mobile phase (overall measurement time of 6 min). The HPLC system was coupled to a UV detector to quantify the concentration of the different model compounds at 280 nm with the help of respective calibration curves.

### Molecular Modeling

The model compound guaiacylglycerol-β-guaiacyl ether (GGE), shown in Figure 2, and the DES ethylene glycol and choline chloride were modeled with CGenFF/CHARMM36 force field.^67,68^

**Figure 2:**
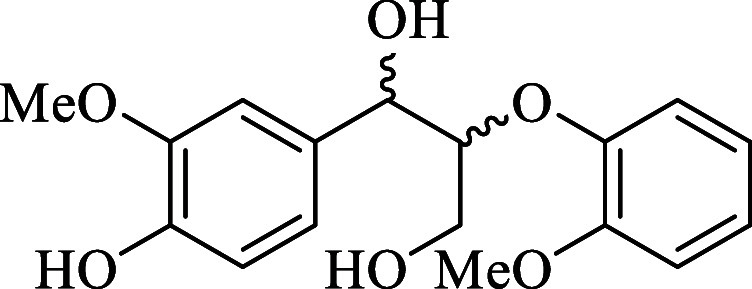
Chemical structure of the Lignin model compound guaiacylglycerol-β-guaiacyl ether (GGE).

The charges for choline chloride were scaled with 0.8 and adapted from Doherty and Acevedo.^69^ The SPC/E water model^70^ was chosen.

### Simulation Details

The molecular dynamics simulations were performed with GROMACS Version 2021.6.^76^ The time step is 1 fs with a cutoff radius of 1.2 nm. Periodic boundary conditions were applied in all three spatial dimensions. The Fast Smooth Particle-Mesh Ewald method^72^ was employed to manage long-range electrostatic interactions.

The molecular simulations for each simulation of the five repetitions can be divided into four steps: (1) creating the initial configuration, (2) energy minimization, (3) equilibration phase, and (4) production phase.

(1) Each simulation system was simulated with five repetitions, starting from different starting configurations. These initial configurations were created with packmol version 20.010.^73^ One molecule of GGE is placed into the approximate (5.5 nm)^3^ simulation box with the number of molecules according to the vol % of the DES-buffer as explained in the experimental section.
(2) In the energy minimization, the steepest descent integrator with a maximal force tolerance of 10 kJ mol^–1^ nm^–1^ was applied.
(3) During the 5 ns equilibration phase, the system stabilizes under temperature and pressure control. Temperature is regulated via the Langevin dynamics stochastic integrator,^71^ while pressure was maintained using the Berendsen barostat.^74^ Equilibration was confirmed by visualization of the properties of interest, like density, temperature, or displacement.
(4) The Parrinello–Rahman barostat^75,76^ was utilized in the production simulations to sample the *NpT* ensemble. The production simulations run for 4 ns with a trajectory output frequency of 1 ps. The production simulation to determine the diffusion coefficient has a trajectory output frequency of 10 fs and runs for 4 ns as well.

### Evaluation of the MD Simulations

The preferential solvation was analyzed by determining Kirkwood–Buff integrals (KBI) *G*_*ij*_^∞^ between compounds *i* and *j*. Thereby, *G*_*ij*_^∞^ is defined as the spatial integral over their pair correlation function g_ij_(*r*)­

1 $$G_{ij}^{\infty }=\int_{0}^{\infty }4\pi r^{2}\left[g_{ij}\left(r\right)‐1\right]dr$$

with *G*_*ij*_^∞^> 0 quantifying an excess of j molecules around i, whereas *G*_*ij*_^∞^ < 0 indicates its deficiency.

The effect of a cosolvent, in this study, the hydrotrope “DES”, on the solute “GGE” molar solubility c_GGE_ can be expressed by

2 $${\left(\frac{\partial \ln c_{GGE}^{*}}{\partial c_{DES}}\right)}_{T,p,c_{GGE}\to 0}=\frac{\left(G_{GGE‐DES}^{\infty }-G_{GGE‐H2O}^{\infty }\right)}{1+c_{DES}\left(G_{DES‐DES}^{\infty }-G_{DES‐H2O}^{\infty }\right)}$$

meaning that it increases when the solute has a higher affinity to the cosolvent as to the primary solvent water “H_2_O”, i.e., (*G*_GGE‑DES_^∞^ − *G*_GGE‑H2O_^∞^) > 0. However, it decreases when the cosolvent tends to self-cluster as (*G*_DES‑DES_^∞^ − *G*_DES‑H2O_^∞^) increases.^77^ Martins et al.^78^ extended eq 2 to account for the primary solvent being already a binary mixture, which we adopt to also study the effect of glycine (Gly)

3 $${kT\left(\frac{\partial \ln c_{GGE}}{\partial c_{DES}}\right)}_{T,p,c_{Gly},\mu_{GGE}=\mu_{GGE}^{0}}=\left[c_{DES}\left(G_{GGE‐DES}^{\infty }-G_{GGE‐H2O}^{\infty }\right)‐\frac{c_{Gly}\left(G_{GGE‐Gly}^{\infty }-G_{GGE‐H2O}^{\infty }\right){\cdot c}_{DES}\left(G_{DES‐Gly}^{\infty }-G_{Gly‐H2O}^{\infty }\right)}{1+c_{Gly}\left(G_{Gly‐Gly}^{\infty }‐G_{Gly‐H2O}^{\infty }\right)}\right]\cdot {\left(\frac{\partial \mu_{DES}}{\partial c_{DES}}\right)}_{T,p,c_{Gly},\mu_{GGE}=\mu_{GGE}^{0}}$$

with the standard deviation of the error propagation $\sigma_{G_{12}^{\infty }-G_{13}^{\infty }}=\sqrt{{\left(\sigma_{G_{12}^{\infty }}\right)}^{2}+{\left(\sigma_{G_{13}^{\infty }}\right)}^{2}}$ .

In this study, the DES serving as a hydrotrope^79^ was introduced into the water (H_2_O) + glycine (Gly) mixture to enhance the dissolution of GGE.

For calculating the KBI for the different pairing of mixture compounds (GGE, water, glycine, EG, choline, and Cl), we use the Python package pykbi^80^ and the analysis code of the Maginn group published on GitHub.^58,81^

The radial distribution function is computed using the GROMACS command *gmx rdf* and corrected with the van der Vegt correction^82^ implemented in pykbi.^80^ The Krüger correction^83^ for the integral is computed according to the implementation in ref 58,81 . To identify the linear regime for extrapolation, two cutoff points were defined. The first cutoff occurs when the slope of the corrected radial distribution function remains constant to within 0.1 over seven successive distance values. The second cutoff is reached once the slope of the Kirkwood–Buff integral deviates by more than 0.1. Further details on the linear regime can be found in the Supporting Information, chapter 7.2 and Figure S27.

To provide a more detailed insight into the microstructural properties of the mixtures, the simulation trajectories were analyzed to derive different properties such as the hydrogen bond number and lifetime, the diffusion coefficient, the solvent accessible surface area (SASA), and the combined distribution function of angle over distance to identify characteristic molecular configurations of dissolved GGE.

The lifetime and number per ps of hydrogen bonds were determined using *gmx hbond* in GROMACS. The overall HB stability is derived from

4 $$\%bonded\;life\;time=\frac{\tau_{existence}}{\tau_{existence}+\tau_{formation}}$$

using the TRAVIS analyzer.^84–86^ The diffusion coefficient was calculated using the Einstein relation. The command *gmx msd* computed the mean squared displacement (msd), while the linear regime of the msd was selected manually. The solvent accessible surface area is quantified as the time average using *gmx sasa*.

The combined distribution function of the angle over the distance from the TRAVIS analyzer^87,88^ is used to identify significant configurations and hydrogen bonds. For hydrogen bonds, the angle between the hydrogen donor and the acceptor needs to occur within 150° to 180°, while the distance between donor must not exceed 250 pm.^89^

## Results and Discussion

### Enzymatic Lignin Depolymerization in Different DES Systems

With the aim to increase the substrate (i.e., lignin) concentration in enzymatic lignin depolymerization reactions using the enzyme cascade shown in Figure 1 (combining two enantiocomplementary Cα dehydrogenases, two enantiocomplementary β-etherases, and an unselective glutathione lyase with a glutathione amide reductase for in situ cofactor regeneration), different DESs were investigated as potential cosolvents in combination with glycine/NaOH buffer. Based on the pH dependence of the cascade enzymes, only nonacidic DES were employed to maintain a pH of 9 in the reaction for optimal cascade performance. The organic cosolvent DMSO was used for comparison since it has previously been described as a suitable cosolvent in enzymatic lignin depolymerization.^31,90^ Moreover, an organosolv lignin from beech wood, provided by the Fraunhofer CBP in Germany, was applied as the lignin polymer.

Initially, the impact of the different DES (based on ethylene glycol, guaiacol, or resorcinol as HBD and betaine or choline chloride as HBA) on the enzyme cascade performance was investigated using a low lignin concentration of 2 g L^–1^ and increasing cosolvent concentrations (Figure 3). As a readout of lignin depolymerization efficiency, the yield of the monoaromatic degradation products SHP and GHP was determined.

**Figure 3:**
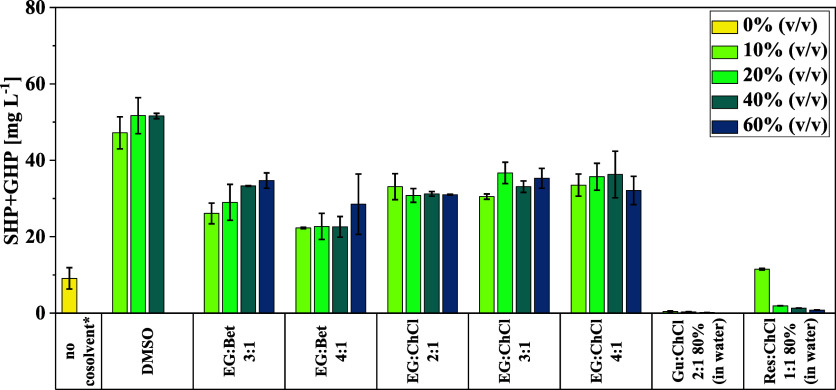
Yield of SHP and GHP in enzymatic lignin depolymerization reactions using different DES and DMSO in varying concentrations for lignin solubilization (Table S3). Reactions were performed at 30 °C and 1000 rpm for 1 h in 250 μL volume containing 400 mM glycine/NaOH buffer, pH 9.0, DMSO or DES concentrations from 10 to 60% (v/v), 2 g L^–1^ lignin, 5 mM NAD^+^, 8 mM GSH, and 0.25 g L^–1^ of each purified enzyme of the cascade. For comparison, a reaction was performed in the absence of a cosolvent, resulting in only partial solubilization of the lignin (no cosolvent*). [EG: ethylene glycol, Bet: betaine, ChCl: choline chloride, Gu: guaiacol, and Res: resorcinol.]

Comparing the different DES, the use of Gu:ChCl 2:1 and Res:ChCl 1:1 turned out to be detrimental for lignin depolymerization using our cascade, as almost no SHP and GHP was obtained even at low DES concentration (Figure 3). This is mainly caused by a deactivating or inhibitory effect of guaiacol and resorcinol on enzyme activity (Supporting Information, chapter 4). In contrast, considerable yields up to 37 mg L^–1^ of SHP and GHP (corresponding to 18.5 mg g^–1^ of lignin) could be attained with the ethylene glycol-based DES. Here, the combination of ethylene glycol and choline chloride seemed to be superior to the DES composed of ethylene glycol and betaine. In the latter case, the SHP and GHP yield increased with increasing DES concentration, which might be indicative of an insufficient solubility of the applied 2 g L^–1^ lignin in the EG:Bet buffer system at low DES concentration. This is further confirmed by the fact that the organosolv lignin used in this study could be dissolved only in up to 40 g L^–1^ in EG:Bet DES, while the three EG:ChCl DES allowed concentrations up to 50 g L^–1^. Moreover, increasing the concentration of the EG:ChCl DES in the reaction system had no significant effect on the yield of SHP and GHP, as similarly high yields were obtained for all tested DES concentrations. Hence, this could open up the possibility for applying even higher lignin concentrations in EG:ChCl-buffer-based reaction systems, as desired for efficient lignin depolymerization. Though a higher SHP and GHP yield was achieved in enzymatic reactions using DMSO as a cosolvent (up to 51.1 mg L^–1^ at 40% DMSO) compared to the tested DES systems, this organic solvent has a clear negative effect on enzyme activity at higher concentrations, as only negligible amounts of SHP and GHP were obtained in reactions using 60% DMSO (Figure 3). In contrast, our enzymatic lignin depolymerization cascade still performed well at 60% DES when using ethylene glycol-based deep-eutectic solvents. This is also confirmed in experiments investigating the impact of the different cosolvents on the activity of the individual enzymes (see Supporting Information, chapter 4). Consequently, the three DES based on EG:ChCl were chosen for further experiments at higher lignin concentrations.

### Influence of Lignin and DES Concentration on SHP and GHP Yield

After establishing EG:ChCl-based DES as suitable cosolvents for enzymatic lignin depolymerization using our cascade, we aimed to explore more lignin:DES concentrations to identify the most suitable DES and to further increase the yield of SHP and GHP. Thus, lignin concentrations up to 30 g L^–1^ were examined while increasing the DES concentration up to 60% (v/v) in the reaction system. Based on a maximum solubility of 50 g L^–1^ lignin in the pure DES systems and the required volumes for adding the six purified enzymes of the cascade as well as glutathione and the cofactor NAD^+^ in buffer, 30 g L^–1^ was the highest lignin concentration that could be investigated. As expected, the volumetric SHP and GHP yields in lignin depolymerization reactions increased with increasing lignin concentration (Figure 4). Interestingly, however, raising the DES concentration while keeping the lignin concentration fixed, did not significantly alter the yield. This implies that the DES concentration does not negatively impact the cascade performance substantially and the obtained SHP and GHP yield is rather a function of lignin concentration in the reaction system. The highest volumetric yield of SHP and GHP was achieved using 50% (v/v) EG:ChCl 4:1 and 20 g L^–1^ lignin (Figure 4D). This condition resulted in 126 mg L^–1^ of SHP and GHP, corresponding to 6.3 mg g^–1^ of lignin (Figure 4D). In contrast, the highest mass-related yield of 32 mg g^–1^ lignin was obtained at 40% (v/v) EG:ChCl 4:1 and 1 g L^–1^ lignin (Figure 5D). Another interesting trend in the data shown in Figures 4 and 5 is the fact that decreasing the proportion of choline chloride in the DES results in an overall higher yield of SHP and GHP. This might be explained by a possible negative impact of choline chloride on the activity and stability of our used enzymes. A destabilizing and inactivating effect of choline chloride on other enzymes, such as laccases, has been described in literature before.^91^ A detailed investigation of the impact of increasing concentrations of the three EG:ChCl DES on the activity of each individual cascade enzyme, however, did not confirm a stronger negative impact for the EG:ChCl 2:1 DES compared to the EG:ChCl 4:1. Instead, relative activities of all enzymes were rather similar (see Supporting Information, chapter 4).

**Figure 4:**
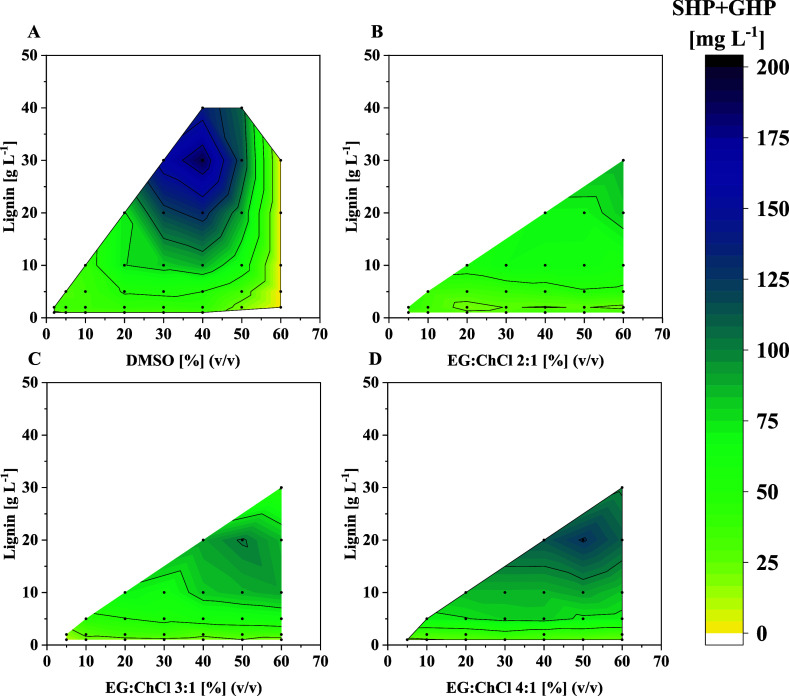
Volumetric yield of SHP and GHP in enzymatic lignin depolymerization reactions at varying lignin and cosolvent (DMSO or EG:ChCl DES) concentration (Table S4). Reactions were performed at 30 °C and 1000 rpm for 1 h in 250 μL volume containing 400 mM glycine/NaOH buffer, pH 9.0, varying DMSO or DES and lignin concentrations, 5 mM NAD^+^, 8 mM GSH, and 0.25 g L^–1^ of each purified enzyme of the cascade.

**Figure 5:**
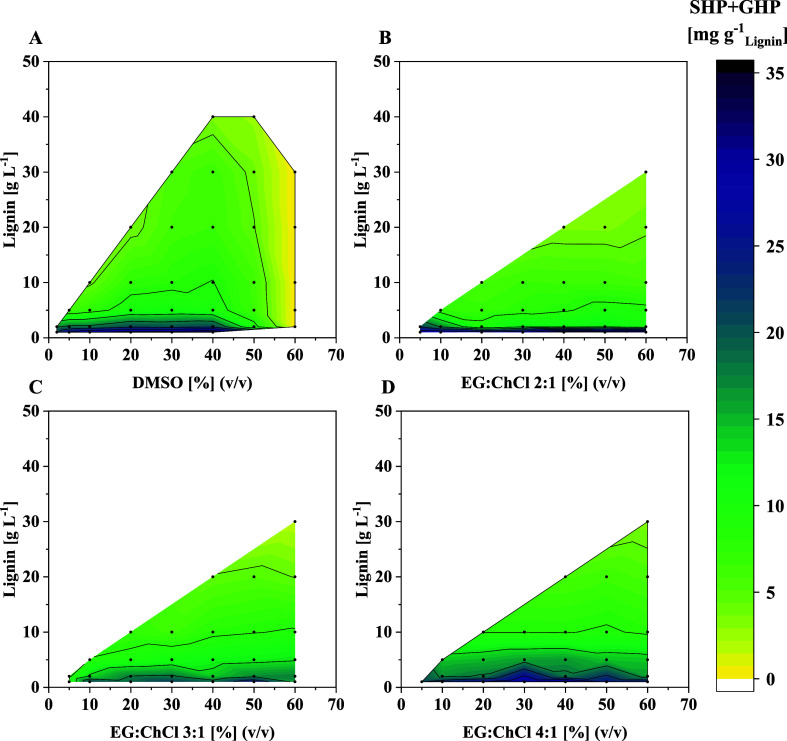
Yield of SHP and GHP per gram of applied lignin in enzymatic lignin depolymerization reactions at varying lignin and cosolvent (DMSO or EG:ChCl DES) concentration (Table S4). Reactions were performed at 30 °C and 1000 rpm for 1 h in 250 μL volume containing 400 mM glycine/NaOH buffer, pH 9.0, varying DMSO or DES and lignin concentrations, 5 mM NAD^+^, 8 mM GSH, and 0.25 g L^–1^ of each purified enzyme of the cascade.

Using DMSO as an organic cosolvent for comparison, a sharp optimum around 30 g L^–1^ lignin and 40% DMSO is observed with a volumetric SHP and GHP yield of 190 mg L^–1^ (corresponding to 6.3 mg g^–1^ lignin) under the applied reaction conditions (Figure 4A). A further increase in DMSO concentration, however, drastically reduces the yield, as observed before (see Figure 4). Despite this higher volumetric yield of SHP and GHP with DMSO compared to the tested DES systems, the respective mass-related yields when using the DES, especially EG:ChCl 4:1, are comparable to those of DMSO. Thus, the highest obtained SHP and GHP yield in relation to the applied lignin concentration that was achieved in reactions with EG:ChCl 4:1 as cosolvent was 32 mg g^–1^ lignin (when using 40% (v/v) DES and 1 g L^–1^ lignin) compared to 32.5 mg g^–1^ lignin for DMSO (when using 20% (v/v) DMSO and 1 g L^–1^ lignin) (Figure 4A + D). Moreover, the same mass-related yield of 6.3 mg g^–1^ lignin was obtained in both cases at the respective optimal conditions for the highest volumetric SHP and GHP yield (Figure 5A + D). Overall, our data highlight that 4:1 EG:ChCl is a promising and green cosolvent for lignin solubilization in combination with our enzymatic lignin depolymerization cascade. However, the question of why EG:ChCl 4:1 outperforms the other EG:ChCl DES with a higher ChCl ratio still remains. Hence, to gain insights into the molecular mechanism of lignin dissolution in EG:ChCl-buffer-based solvent systems, detailed MD simulation studies were performed using GGE as the lignin model compound. Though GGE will likely not represent the full structural diversity present in the employed organosolv lignin K053, it was chosen as an ideal model for the structure that is targeted and cleaved by our enzyme cascade for lignin depolymerization.

### Solubility of GGE in Water + DES + Glycine Mixtures

The following sections present a detailed MD analysis of the preferential solvation of the lignin model compound GGE and the structural features of EG:ChCl-based DES–glycine buffer mixtures. We thereby explore different DES:buffer ratios (12.5%, 25%, and 50%) and varying EG:ChCl molar ratios of the DES (4:1, 3:1, and 2:1). Figure 6 plots the experimental solubility of GGE versus the solvent molecular weight for each system.

**Figure 6:**
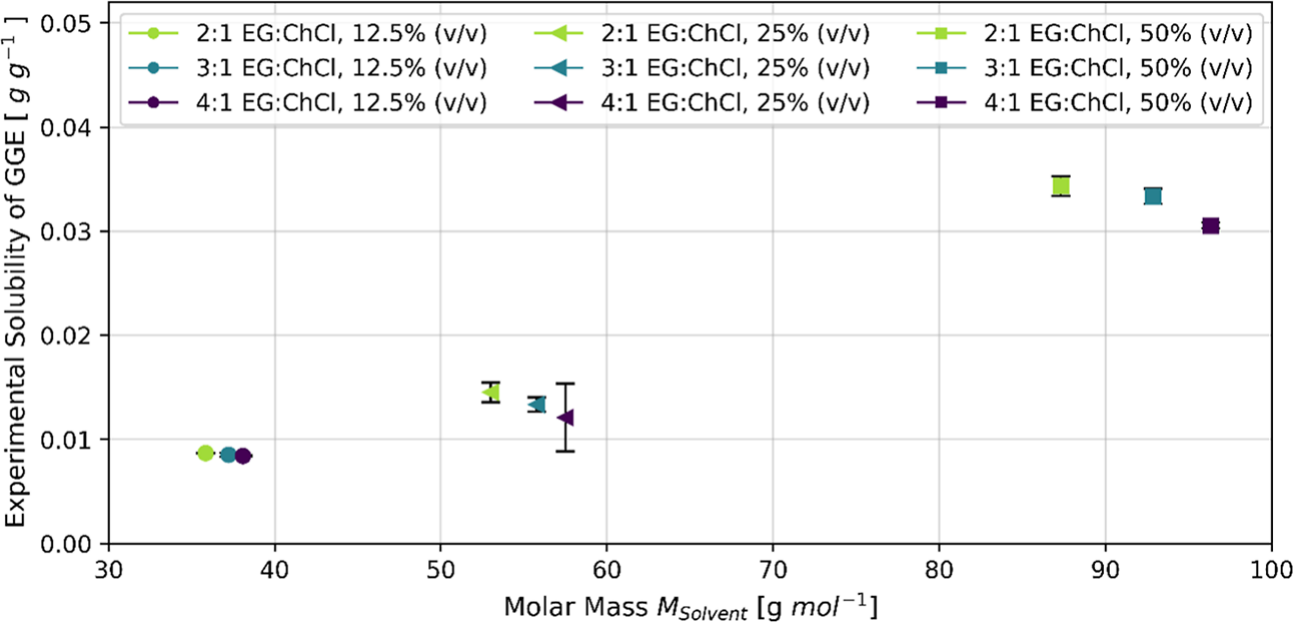
Experimental solubility of the model compound GGE as a function of solvent molar mass at EG:ChCl molar ratios of 2:1, 3:1, and 4:1 and for three solvent compositions of DES:glycine-water buffer: 12.5% (v/v), 25% (v/v), and 50% (v/v) DES.

The solubility of GGE increases with the molar mass of the solvent, with the latter being a direct result of the higher DES concentration in the solvent. This is consistent with findings in literature^79^ that for ChCl-based DES, the lignin solubility decreases with increasing water content due to the favorable interactions of water with the Cl^–^ anion. Interestingly, for a constant DES:water ratio, 2:1 EG:ChCl yields the highest GGE solubility, whereas the solubility decreases with an increasing EG to ChCl ratio. This is in contrast to the studies on the solubility of lignin monomeric model compounds in different DES by Soares et al.^35^ in which they observed that lignin solubility increases with a higher HBD ratio. However, in their study on Kraft lignin solubility in DES, Sosa et al.^79^ could not find a significant effect of HBA-HBD ratio for the EG:ChCl DES.

### Kirkwood–Buff Integrals

To get a first insight into the preferential solvation in the complex solvent mixtures consisting of water, DES, and glycine buffer, the Kirkwood–Buff integrals (KBI) of GGE with the solvent compounds were analyzed. The DES serves as a hydrotrope in aqueous solution,^79^ whereas the primary solvent is a binary mixture of water with the buffer compound glycine (see eq 3). The most significant systems in this study are 2:1 EG:ChCl with the highest lignin solubility and 4:1 EG:ChCl with the highest GHP + SHP yield. The terms from eq 3 that affect the solubility of GGE in the DES + water + glycine mixture are discussed separately.

According to eq 3, the main impact on the solubility of the solute is the positive solute-hydrotrope (DES) preferential interactions relative to solute–water interactions indicated with *c*_DES_(*G*_GGE‑DES_^∞^–*G*_GGE‑H2O_^∞^) > 0. Figure 7 shows a positive difference of GGE-EG, which indicates that EG is more favorable around GGE than water and, thereby, increases the solubility of GGE. However, GGE is slightly more preferential to being surrounded by water than choline chloride, which reduces the solubility. As the DES content increases, the beneficial GGE-EG interactions become increasingly pronounced. In the 2:1 EG:ChCl mixture, the adverse influence of the relatively weak GGE-ChCl interaction is attenuated, whereas in the 4:1 system it intensifies. A direct comparison of both 50% (v/v) DES mixtures shows that GGE-EG interactions dominate, while the unfavorable GGE-ChCl interactions exert only a marginal effect, which supports the higher experimentally observed solubility of GGE in 2:1 composition.

**Figure 7:**
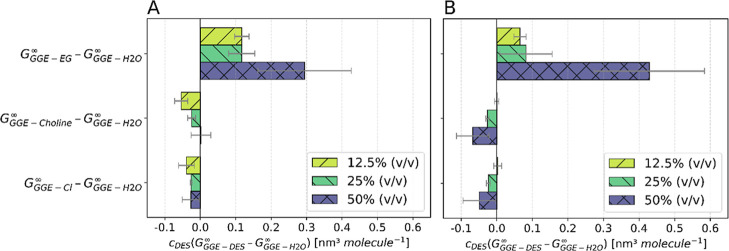
Differences of the Kirkwood–Buff integrals *c*_DES_(*G*_GGE‑DES_^∞^–*G*_GGE‑H2O_^∞^) of (A) 2:1 and (B) 4:1 EG:ChCl for three solvent compositions: 12.5% (v/v), 25% (v/v), and 50% (v/v) DES in glycine-water buffer.

The differences shown in Figure 8 are positive, indicating the favorable solute–salt interaction. At 4:1 EG:ChCl, increasing the DES fraction promotes preferential glycine coordination around GGE, while for 2:1 EG/ChCl, higher DES content shifts the balance toward enhanced GGE-water interactions. The comparison at 50% (v/v) DES shows that the small absolute difference at 2:1 EG:ChCl reduces the negative impact on the solubility, leaving the (*G*_GGE‑EG_^∞^–*G*_GGE‑H2O_^∞^) difference to dominate. The strong negative impact at 4:1 EG:ChCl advocates the lower solubility of GGE in the mixture.

**Figure 8:**
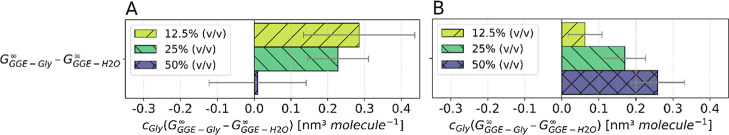
Differences of the Kirkwood–Buff integrals *c*_Gly_(*G*_GGE‑Gly_^∞^–*G*_GGE‑H2O_^∞^) of (A) 2:1 and (B) 4:1 EG:ChCl for three solvent compositions: 12.5% (v/v), 25% (v/v), and 50% (v/v) DES in glycine-water buffer.

To identify preferential interactions within the solvent, the KBI of the DES, water, and buffer compounds are computed, and the terms according to eq 3 are shown in Figures 9 and 10.

**Figure 9:**
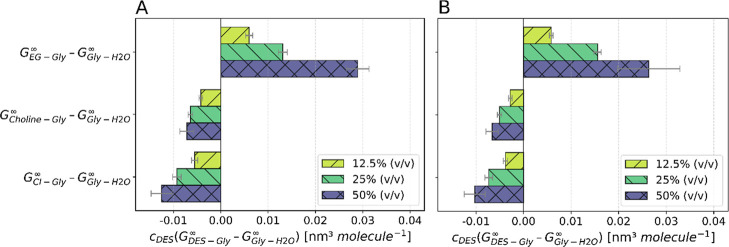
Differences of Kirkwood–Buff integrals c_DES_(*G*_DES‑Gly_^∞^–*G*_Gly‑H2O_^∞^) of (A) 2:1 EG:ChCl and (B) 4:1 EG:ChCl with different DES:buffer ratios: 12.5% (v/v), 25% (v/v), and 50% (v/v).

**Figure 10:**
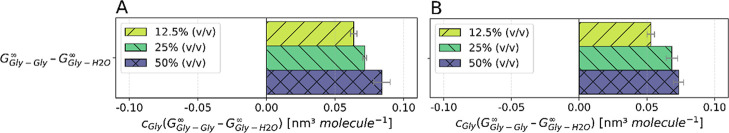
Differences of Kirkwood–Buff integrals c_Gly_(*G*_Gly‑Gly_^∞^–*G*_Gly‑**H**2O_^∞^) of (A) 2:1 EG:ChCl and (B) 4:1 EG:ChCl with different DES:buffer ratios: 12.5% (v/v), 25% (v/v), and 50% (v/v).

As the DES:buffer ratio increases, the preferential interactions between EG-glycine relative to glycine-water become more pronounced, thereby exerting a reducing effect on the solubility of GGE. Conversely, glycine-water interactions remain favored over ChCl-glycine associations, reversing the sign in eq 3 accordingly, predicting a positive contribution to the solubility. With an increasing DES/buffer ratio, the absolute difference increases, meaning that the impact–negative or positive–on the solubility rises. Nevertheless, the observed KBI differences are very small and thus, exert only a marginal influence on the solubility.

The last term is the denominator 1+*c*_Gly_(*G*_Gly‑Gly_^∞^–*G*_Gly‑H2O_^∞^). The self-association of glycine to the glycine-water association causes the deviation from 1 without any effect on the sign of the fraction.

Glycine preferentially engages with itself rather than with water, as indicated by a positive difference in their Kirkwood–Buff integrals. A larger positive difference increases the denominator of eq 3, thereby improving the solubility of GGE. This advantageous effect is slightly stronger in the 2:1 EG:ChCl system than in 4:1 composition.

### Hydrogen Bond Analyses

Hydrogen bonds are identified as having an important impact on solubility and solvent behavior.^78,92^ We evaluate HB interactions using three metrics: the total number of HB per picosecond between the DES components and water, the compound-wise lifetimes, and the stability of HB indicated by the percentage bonded lifetime during which the HB existed in relation to the formation time.

The hydrogen bond analysis in Figure 11 shows that increasing the DES content leads to a larger number of hydrogen bonds per picosecond between water and DES, which correlates with higher solubility. In detail, ethylene glycol exhibits its maximum HB count per ps at 4:1 EG:ChCl ratio—correlating the maximal observed reaction yield—whereas the maximal HB count per ps of water and HBA occurred at a 2:1 ratio, in line with the solubility trends. The absolute number of hydrogen bonds per ps between HBD and water is higher due to the ratio of HBD:HBA of DES.

**Figure 11:**
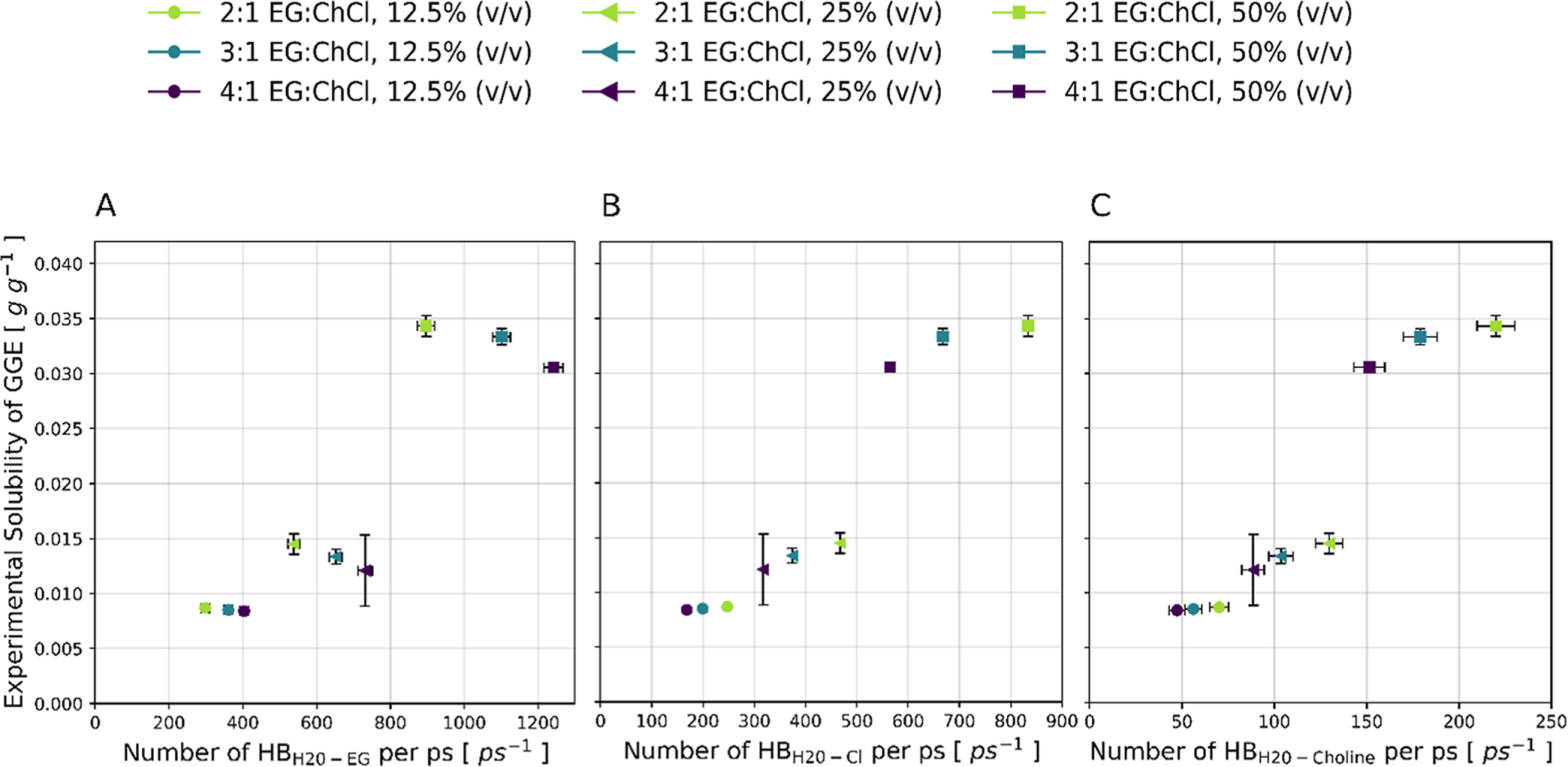
Experimental solubility over the number of HB per ps between (A) H_2_O–EG, (B)­H_2_O–Cl, and (C) H_2_O–choline.

As 2:1 EG:ChCl mixtures exhibit the highest solubility and 4:1 the maximal reaction yield at both 50% (v/v) DES, all subsequent analyses will focus on these two systems.

Figure 12 shows the hydrogen bond lifetimes of all intra- and intermolecular combinations in the GGE-DES-buffer systems. The HB lifetimes of GGE-DES, EG-choline, and DES-water are higher at 2:1 EG:ChCl than at 4:1 EG:ChCl, indicating the importance of these HBs for the solubility. Chloride forms long-lived hydrogen bonds, especially with water. Together with the positive values of the Cl–H_2_O KBI (see Table S6), it clearly illustrates the amphiphilic character of the DES and its crucial role in lignin dissolution. Glycine builds rather short hydrogen bonds.

**Figure 12:**
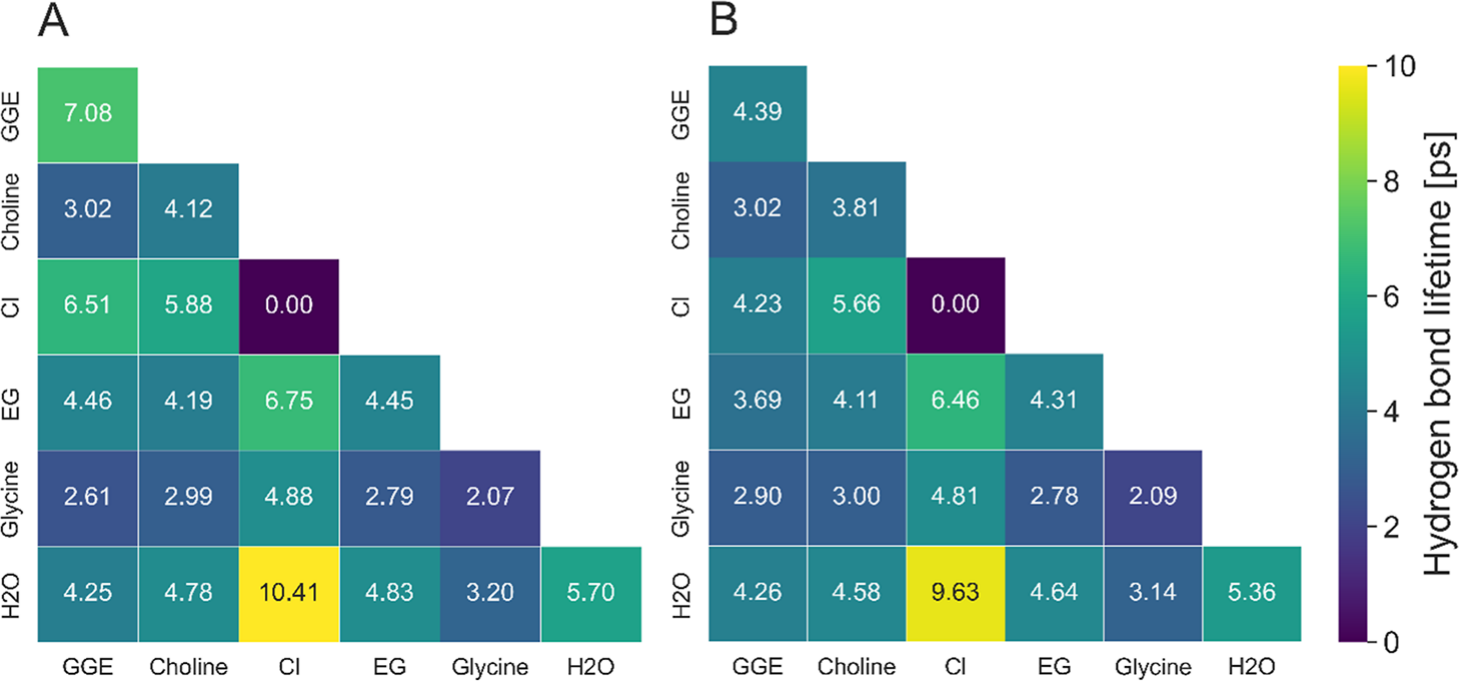
Intermolecular and intramolecular hydrogen bond lifetimes in [ps] of all compounds in the GGE-DES-buffer mixtures of (A) 2:1 EG:ChCl and (B) 4:1 EG:ChCl composition.

Next to the HB lifetime, the stability of hydrogen bonds is investigated with the ratio of existence time to the sum of existence and formation time (eq 4).

The hydrogen bond stability analysis in Figure 13 shows that the overall %bonded lifetimes are greater in the 2:1 EG:ChCl than in the 4:1 DES composition, which indicates the positive impact on the solubility. Furthermore, chloride anions display their highest HB stability at an EG:ChCl ratio of 2:1. These findings confirm that chloride anions are the most persistent HB donors in the system.^86^ When comparing the two systems, at an EG:ChCl ratio of 4:1, GGE-EG exhibits a higher %bonded lifetime, which correlates with the observed increase in reaction yield. This would suggest that the overall lower stability of HB enhances the reaction and increases the reaction yield.

**Figure 13:**
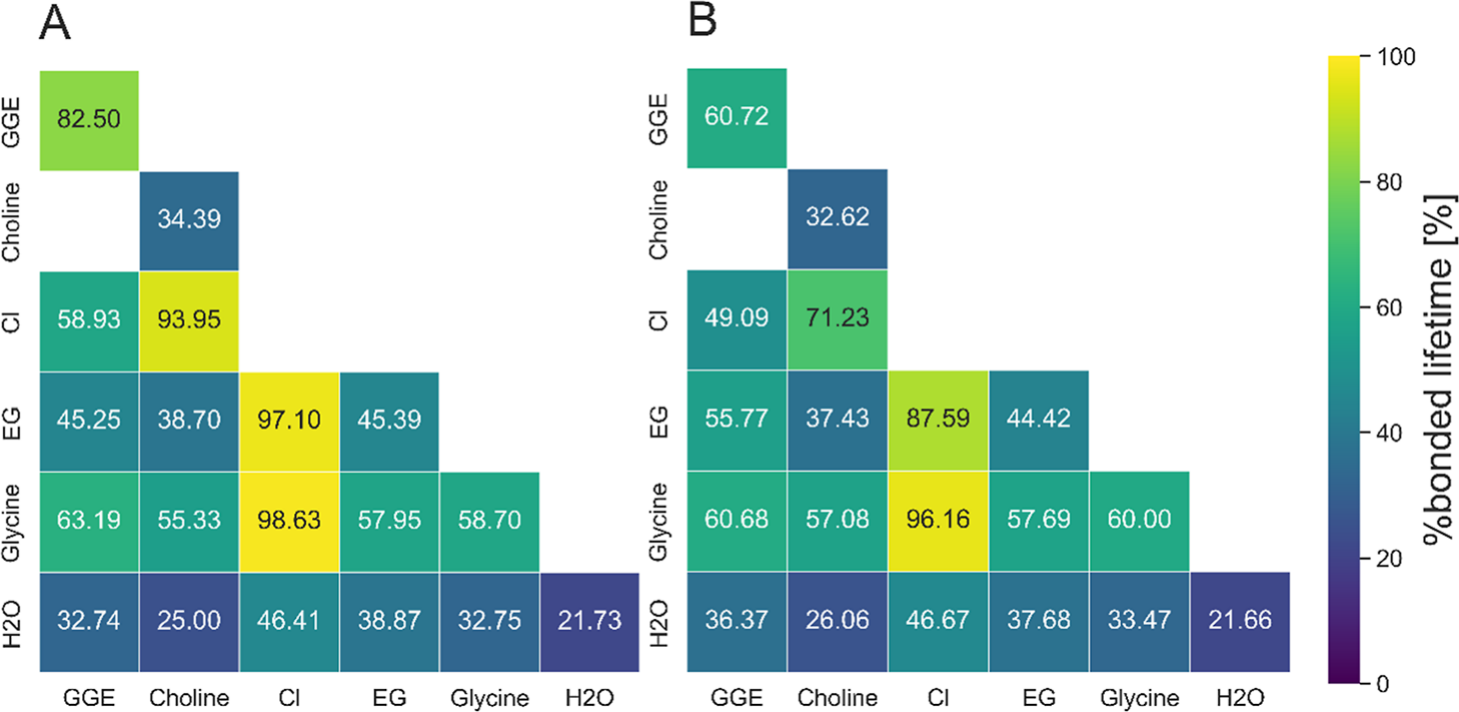
Percentage bonded lifetime of intermolecular and intramolecular hydrogen bond of all compounds in the GGE-DES-buffer mixture of (A) 2:1 EG:ChCl and (B) 4:1 EG:ChCl composition.

General findings of the HB analysis of the number, the lifetime, and the stability of hydrogen bonds show that longer and stronger HB between GGE and the solvent and within the solvent lead to the higher solubility of the 2:1 EG:ChCl composition. The number of HB between ChCl and water, as well as the more stable HB between ChCl within the solvent, underlays the important role of HBA to the solubility of the model compound, which agrees with the results of the Kirkwood–Buff integrals. Interestingly, the Cl^–^–H_2_O interaction has the longest HB but is not significantly stable, whereas Cl^–^-glycine have rather short but stable HB.

Apparently, the shorter and weaker HB in the 4:1 EG:ChCl mixture increased the reaction yield. The high number of EG around GGE agrees with the KBI results, but the short and midstable hydrogen bonds indicate a positive effect to the reaction yield.

The different lifetimes and stabilities of HB within GGE require a detailed analysis of the intramolecular HB analysis and configurations of GGE along with the dynamic behavior of the systems.

### Dynamics and Configuration of GGE

Detailed insights into the configuration of GGE during the simulation support the understanding of the ability of the enzymatic reaction. The β-*O*-4 bond must be cleaved as part of the enzymatic cascade. Therefore, the intermolecular HB around the β-*O*-4 bond and the configuration of the GGE are investigated in more detail.

Figure 14 shows the combined distribution function of the angle over the distance of two hydroxyl groups (O2–H2 and O3–H3) with the keto group, O1, in the ring.

**Figure 14:**
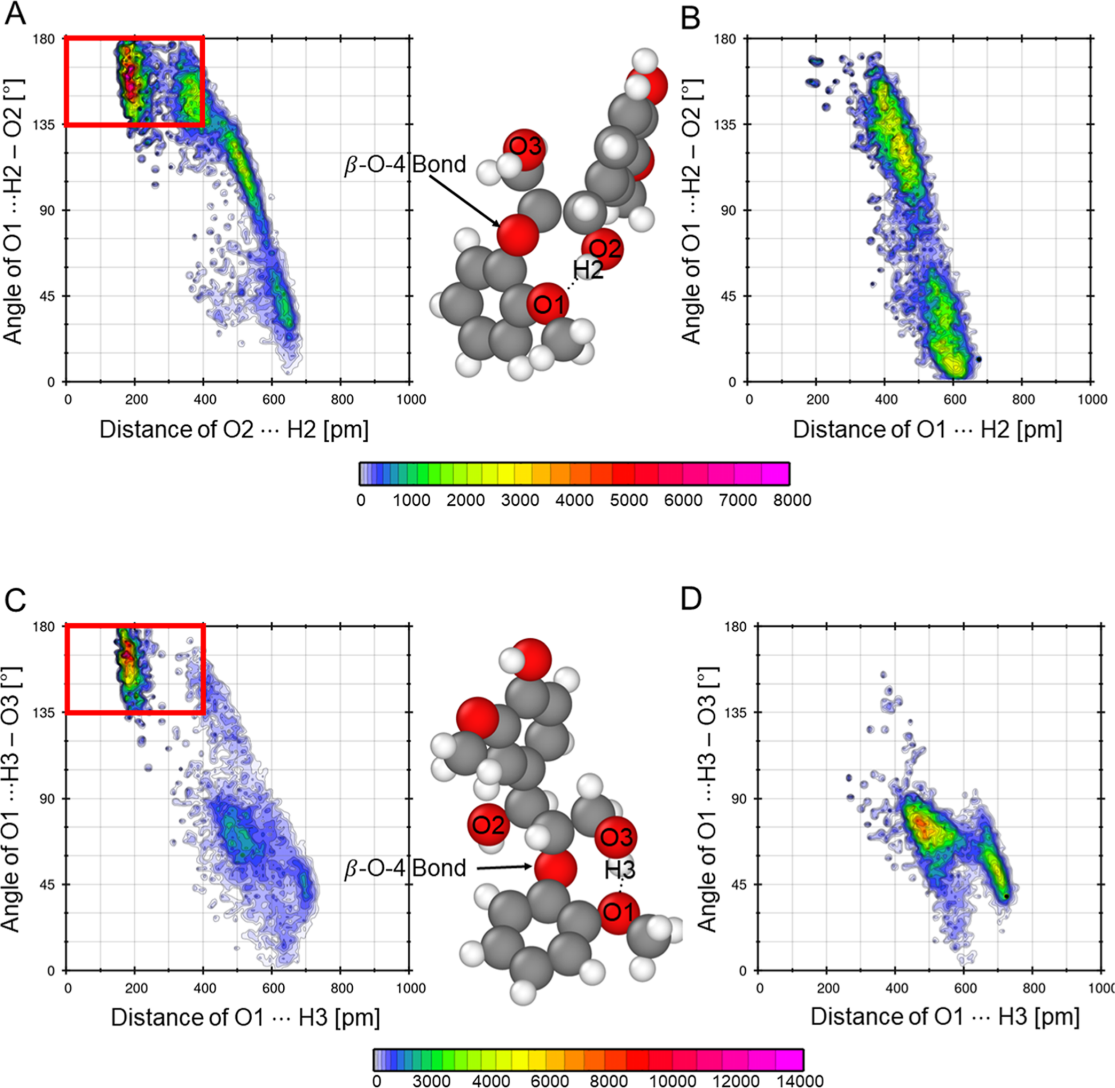
Combined distribution functions of GGE in (A) 2:1 EG:ChCl of O1···H2 and (B) 4:1 EG:ChCl of O1···H2, (C) 2:1 EG:ChCl of O1···H3 and (D) 4:1 EG:ChCl of O1···H3 at 50% (v/v) DES to identify intramolecular hydrogen bond around the β-*O*-4 bond. The red box indicates the geometric criteria for HB.

In the 2:1 EG:ChCl mixture, hydrogens H2 and H3 engage in medium-strong to strong hydrogen bonds with the ring’s keto oxygen O1, effectively spanning the β-*O*-4 bond. In contrast, the 4:1 EG:ChCl system lacks these specific intramolecular interactions, exhibiting only weaker, longer–range interactions between the hydroxyl groups at different angles. The intramolecular HB of the 4:1 EG:ChCl composition that is demonstrated in Figures 12 and 13 is built between O2–H2 and O3–H3, as shown in Figure 15B.

**Figure 15:**
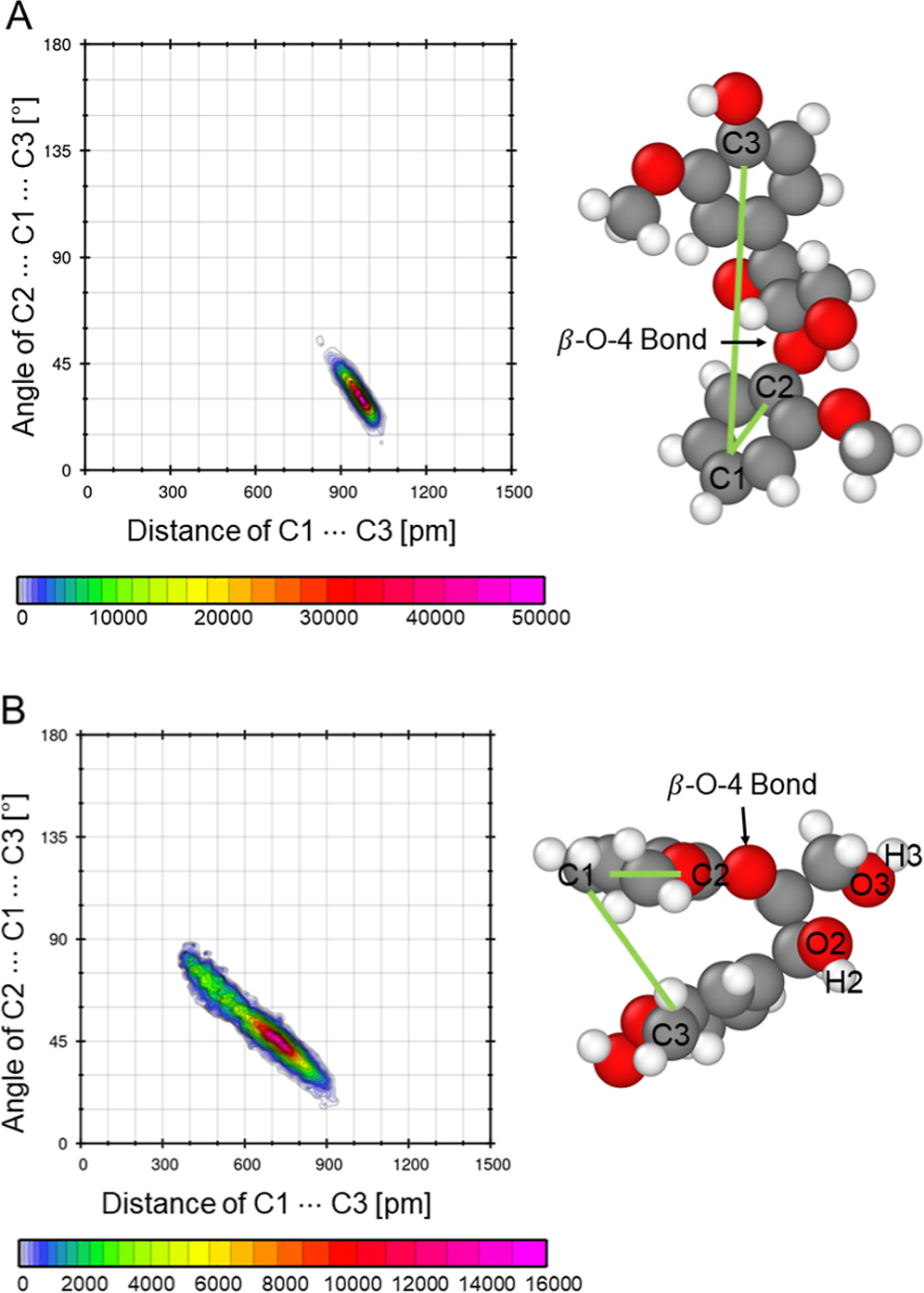
Combined distribution functions of the aromatic rings in GGE in (A) 2:1 EG:ChCl and (B) 4:1 EG:ChCl at 50% (v/v) DES.

All combined distribution functions in Figure 14 show the occurrence of angle–distance combinations at around 500 pm and varying angles from 60°–120°. These occurrences indicate fluctuations in the GGE conformation. To evaluate the dynamics of the GGE systems, the diffusion coefficient and the SASA are listed in Table 2.

**Table 2:** Diffusion Coefficient and Solvent Accessible Surface Area (SASA) of GGE in 2:1 EG:ChCl and 4:1 EG:ChCl in 50% (v/v) DES

system	diffusion coefficient [m^2^ *s* ^–1^]	SASA [nm^3^]
2:1 EG:ChCl	4.2741 · 10^–11^ ± 1.0495 · 10^–11^	5.69566 ± 0.0407
4:1 EG:ChCl	7.2216 · 10^–11^ ± 0.6592 · 10^–11^	5.62306 ± 0.0524

In the 2:1 EG:ChCl system, the hydrogen bonds give rise to an open, widespread GGE configuration and a correspondingly higher SASA. The longer and stronger intermolecular HB interactions correlate with a reduced diffusion coefficient and enhanced solubility, but they also effectively shield the ring’s keto oxygen (O1), limiting its availability for reaction to cut the β-*O*-4 bond.

In contrast, the 4:1 EG:ChCl systems lack intramolecular hydrogen bonds between the hydroxyl group and the keto oxygen at the ring, leaving both the keto oxygen (O1) and the hydroxyl group (O2–H2) fully exposed and therefore more reactive.

The SASA also varies due to the configuration of the two aromatic rings of GGE. The combined distribution functions to identify the angles and distances of the two aromatic rings are shown in Figure 15.

A closer inspection of the aromatic rings of GGE in 4:1 EG:ChCl DES solution shows that although they remain in close proximity, they undergo pronounced conformational fluctuations. Additionally, GGE exhibits a significantly higher diffusion in 4:1 EG:ChCl. These dynamics seem to enhance efficient β-*O*-4 bond cleavage. In contrast, the angle and distance of the aromatic rings of GGE in 2:1 EG:ChCl stay stable, indicating less configurational fluctuations.

## Conclusion

Overall, our work highlights the benefit of using EG:ChCl-based DES in β-etherase-catalyzed lignin depolymerization reactions to increase lignin solubility while maintaining a high enzyme cascade efficacy. This enabled the use of elevated lignin concentrations in depolymerization reactions, thus significantly increasing the volumetric yield of desired monoaromatic degradation products. Even though DMSO turned out to be slightly more efficient in lignin dissolution than the EG:ChCl-based DES, it proved more detrimental in terms of enzyme inactivation and is usually more challenging in later downstream processing steps.

Interestingly, a marked difference in enzyme cascade performance for lignin depolymerization was observed, depending on the EG:ChCl ratio of the applied DES, which did not correlate with the respective impact of the DES on enzyme activity. Using detailed MD analyses to investigate the interactions between the different components of the solvent mixture and GGE as a lignin model compound, important insights into the molecular determinants affecting lignin dissolution and enzymatic depolymerization could be gained. Thus, Kirkwood–Buff integrals were employed to quantify solute–solvent affinities, revealing that ethylene glycol exhibits a pronounced affinity for the lignin model compound GGE, which correlates with enhanced GGE solubility. Moreover, we have demonstrated that the buffer component glycine exerts a positive effect on the solubility. Intermolecular hydrogen bond analysis highlighted the key role of long-lived, stable hydrogen bonds of chloride, especially with water, illustrating the crucial role of the amphiphilic character of the DES in lignin dissolution. The 2:1 EG:ChCl mixture at 50% (v/v) DES provides the highest GGE solubility, which correlates with an open, extended intramolecular conformation stabilized by intramolecular hydrogen bonds. In contrast, in the 4:1 EG:ChCl composition at 50% (v/v) DES, the model compound adopts a more flexible intramolecular structure with weaker hydrogen bond interactions and a higher diffusion coefficient, which might explain the higher observed depolymerization efficiency for lignin. Thus, our work underscores the critical role of understanding the complex solvent–solute interactions and their effect on solute conformations and dynamics in order to provide guidelines for the rational design of DES systems tailored to the enzymatic lignin depolymerization cascade.

## Supplementary Material



## Notes

Additional figures and tables regarding protein production and purification, the solubility of the lignin model compounds; the yield of SHP and GHP from enzymatic lignin depolymerization and the impact of DMSO and the DES on enzyme activity; HPLC analysis and calibration curves; chemical synthesis of model compounds; molecular dynamics information (PDF)

## Abbreviations

Bet: betaine
ChCl: choline chloride
Cl: chloride
CV: column volumes
DES: deep eutectic solvent
DMSO: dimethyl sulfoxide
EG: ethylene glycol
GAR: Glutathione amide reductase
GGE: guaiacylglycerol-β-guaiacyl ether
GHP: guaiacyl hydroxy propanone
GSH: glutathione
GSSG: glutathione disulfide
Gu: guaiacol
HB: hydrogen bond
HBA: hydrogen bond acceptor
HBD: hydrogen bond donor
HPLC: high-performance liquid chromatography
IPTG: isopropyl β-D-1-thiogalactopyranoside
KBI: Kirkwood–Buff integral
msd: mean squared displacement
MD: molecular dynamics
NAD^+^: nicotinamide adenine dinucleotide (oxidized)
NADH + H^+^: nicotinamide adenine dinucleotide (reduced)
Ni-NTA: nickel-nitriloacetic acid
PAGE: polyacrylamide gel
Res: resorcinol
SASA: solvent accessible surface area;
SHP: syringyl hydroxy propanone
SDS: sodium dodecyl sulfate
TB: terrific broth
TFA: trifluoroacetic acid
