# Supplementary material for: Efficient Lignin Dissolution and Enzymatic Depolymerization in Ethylene Glycol-Based Deep Eutectic Solvent Mixtures: Integrated Experimental and Molecular Dynamics Study
Source: J Phys Chem B. 2026 Feb 11;130(8):2361–76. doi: 10.1021/acs.jpcb.5c07451 (PMC12951573; doi:10.1021/acs.jpcb.5c07451)
Supplement: Supplementary file 1 [file jp5c07451_si_001.pdf]

Supporting Information

Efficient lignin dissolution and enzymatic depolymerization in ethylene glycol-based deep eutectic solvent mixtures: Integrated experimental and molecular dynamics study

Julian Schilke [a,†], *Miriam Sprick* [b,†], *Eduardo Schneider* [b], *Josué Arturo Ledezma Fierro* [d], *Alexandre Pradal* [d], *Gabriele Raabe* [b,c,*], *Anett Schallmey* [a,c,*]

[a] Institute of Biotechnology, Bioinformatics and Biochemistry, Technische Universität Braunschweig, Germany

[b] Institute of Thermodynamics, Technische Universität Braunschweig, Hans-Sommer-Straße 5, 38106 Braunschweig, Germany

[c] Center of Pharmaceutical Engineering, Technische Universität Braunschweig, Franz-Liszt-Strasse 35a, 38106 Braunschweig, Germany

[d] Institut Parisien de Chimie Moléculaire, Sorbonne Université, 4 place Jussieu, 75005 Paris, France

† J. Schilke and M. Sprick contributed equally to this paper

* corresponding authors: g.raabe@tu-braunschweig.de, a.schallmey@tu-braunschweig.de

Contents

1. Protein production and SDS-PAGE 3
2. Solubility tests for lignin model compounds 4
3. Yield of SHP and GHP from enzymatic lignin depolymerization 5
3.1 Yield of SHP and GHP in enzymatic lignin depolymerization using different cosolvents ... 5
3.2 Yield of SHP and GHP in enzymatic lignin depolymerization at varying lignin and cosolvent concentrations 6
3.3 HPLC chromatograms of lignin depolymerization reaction 10
3.4 GC-MS of SHP and GHP from enzymatic lignin depolymerization 11
4. Enzyme activity in the presence of DMSO and DES 13
4.1 Activity of LigD and LigN in the presence of DMSO and DES 13
4.2 Activity of LigE-NA and LigF-261 in the presence of DMSO and DES 15
4.3 Activity of TtGAR in the presence of DMSO and DES 17
4.4 Activity of $NaGST_{Nu}$ in the presence of DMSO and DES 19
5. Synthesis of model compounds 21
5.1 Chemicals 21
5.2 Synthesis of 4-(2-(3,4-dimethoxyphenyl)-2-oxoethoxy)-3-methoxybenzaldehyde 21
5.3 Synthesis of 4-((1-(3,4-dimethoxyphenyl)-3-hydroxy-1-oxopropan-2-yl)oxy)-3-methoxybenzaldehyde, (VN-VG) 22
5.4 Synthesis of N5-((R)-1-((carboxymethyl)amino)-3-((2-(3,4-dimethoxyphenyl)-2-oxoethyl)thio)-1-oxopropan-2-yl)-L-glutamine, GS-βVG 23
6. Calibration curves 25
6.1 HPLC calibration curves 25
6.2 CLARIOstar calibration curves 27
7. Molecular dynamics information 29
7.1 Number of molecules of the MD systems 29
7.2 Code process of KBI calculation 30
7.3 Kirkwood-Buff Integral values 32
References 33

1. Protein production and SDS-PAGE

Table S1: Produced enzymes with their respective Accession number, molecular weight, and calculated excitation coefficient at 280 nm with ProtParam[1], as well as their yield.

**Protein**	**Accession number**	**M [g mol^{-1}]**	**ε_{280nm} [L mol^{-1} cm^{-1}]**	**Yield [mg L^{-1}]**
LigN	WP_014077904	35089.80	23950	455
LigD	WP_014075190	34506.94	24410	329
LigE-NA	WP_011446047	33285.67	53400	252
LigF-261	WP_104831261	31024.32	52940	420
NaGST$_{Nu}$	WP_011446237	34245.60	57870	333
TtGAR	WP_153973747	51171.36	41370	280

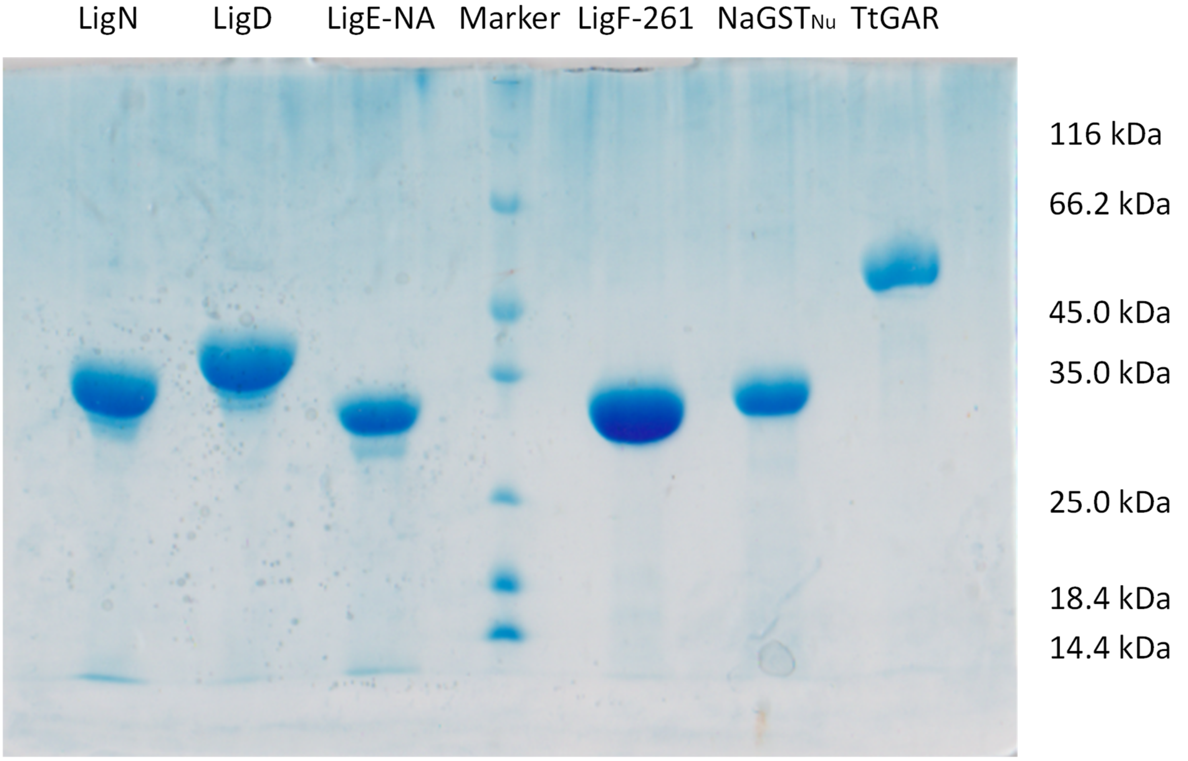

Figure S1: SDS-PAGE of the purified enzymes, which were used for the lignin depolymerization reactions. From left to right: LigN, LigD, LigE-NA, Protein-Marker, LigF-261, NaGST$_{Nu}$, and TtGAR.

2. Solubility tests for lignin model compounds

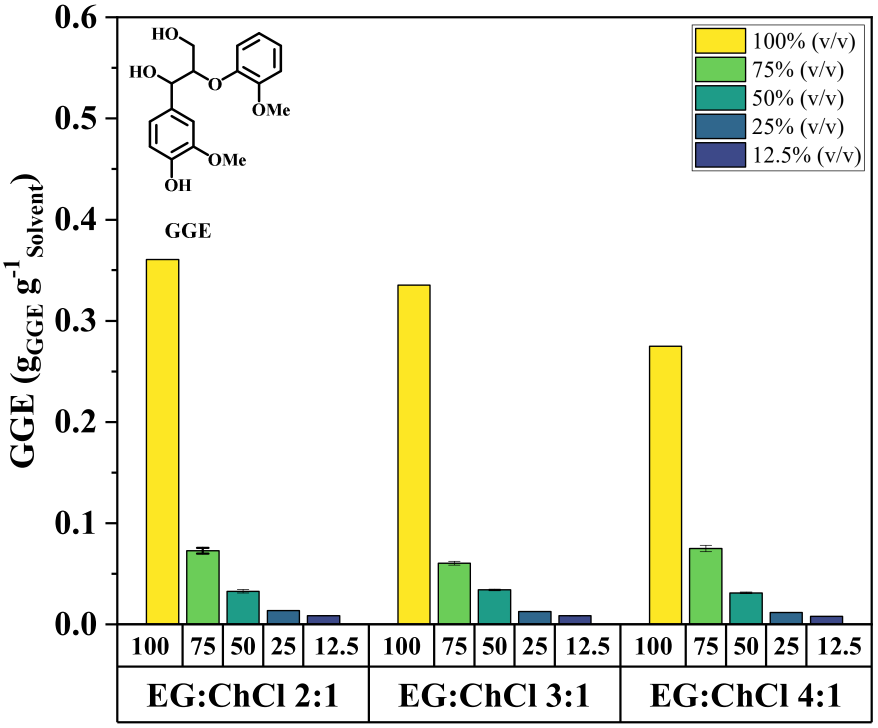

Figure S2: Solubility of the lignin model compounds GGE per gram of DES-buffer system based on HPLC measurements. The GGE concentration was quantified via a calibration curve (Fig. S20)

Table S2: DES-buffer systems, their respective densities, and solubilized model compound amounts.

EG:ChCl	Buffer	pH	DES [%] (v/v)	Density [g/mL]		GGE [$g/g_{Solvent}$]	
2:1			100	1.127	± 0.000	0.360754	± 0
2:1	Glycin	9	75	1.113	± 0.001	0.072840	± 0.002860
2:1	Glycin	9	50	1.079	± 0.002	0.032821	± 0.001630
2:1	Glycin	9	25	1.046	± 0.001	0.013527	± 0.000032
2:1	Glycin	9	12.5	1.028	± 0.001	0.008559	± 0.000038
3:1			100	1.123	± 0.000	0.335466	± 0
3:1	Glycin	9	75	1.114	± 0.001	0.060451	± 0.001840
3:1	Glycin	9	50	1.096	± 0.001	0.034109	± 0.000653
3:1	Glycin	9	25	1.056	± 0.000	0.012720	± 0.000032
3:1	Glycin	9	12.5	1.029	± 0.001	0.008425	± 0.000051
4:1			100	1.122	± 0.001	0.274828	± 0
4:1	Glycin	9	75	1.116	± 0.001	0.074959	± 0.003070
4:1	Glycin	9	50	1.085	± 0.002	0.031135	± 0.000695
4:1	Glycin	9	25	1.051	± 0.001	0.011761	± 0.000101
4:1	Glycin	9	12.5	1.037	± 0.001	0.007955	± 0.000173

3. Yield of SHP and GHP from enzymatic lignin depolymerization

3.1 Yield of SHP and GHP in enzymatic lignin depolymerization using different cosolvents

Table S3: Volumetric yield of SHP and GHP via enzymatic lignin depolymerization, using different cosolvents and constant lignin concentration. Reactions marked with the sign "*" show reactions, were the lignin was only partially solubilized.

Cosolvent	Cosolvent [%] (v/v)	Lignin [g L^{-1}]	SHP+GHP [mg L^{-1}]
**No cosolvent***	0	2	9.1±2.8
**DMSO**	10	2	46.3±4.2
**DMSO**	20	2	56.1±4.7
**DMSO**	40	2	51.5±0.7
**DMSO**	60	2	0.0±0.0
**EG:Bet 3:1**	10	2	26.1±2.7
**EG:Bet 3:1**	20	2	29.0±4.7
**EG:Bet 3:1**	40	2	33.3±0.1
**EG:Bet 3:1**	60	2	34.7±2.0
**EG:Bet 4:1**	10	2	22.3±0.2
**EG:Bet 4:1**	20	2	22.7±3.4
**EG:Bet 4:1**	40	2	22.6±2.7
**EG:Bet 4:1**	60	2	28.5±7.9
**EG:ChCl 2:1**	10	2	33.1±3.4
**EG:ChCl 2:1**	20	2	30.8±1.8
**EG:ChCl 2:1**	40	2	31.2±0.6
**EG:ChCl 2:1**	60	2	31.0±0.1
**EG:ChCl 3:1**	10	2	30.5±0.7
**EG:ChCl 3:1**	20	2	36.7±2.8
**EG:ChCl 3:1**	40	2	33.1±1.5
**EG:ChCl 3:1**	60	2	35.3±2.6
**EG:ChCl 4:1**	10	2	33.5±2.9
**EG:ChCl 4:1**	20	2	35.7±3.5
**EG:ChCl 4:1**	40	2	36.3±6.1
**EG:ChCl 4:1**	60	2	32.1±3.7
**Gu:ChCl 2:1 80% (in water)**	10	2	0.4±0.1
**Gu:ChCl 2:1 80% (in water)**	20	2	0.3±0.1
**Gu:ChCl 2:1 80% (in water)**	40	2	0.2±0.0
**Gu:ChCl 2:1 80% (in water)**	60	2	0.0±0.0
**Res:ChCl 1:1 80% (in water)**	10	2	11.4±0.2
**Res:ChCl 1:1 80% (in water)**	20	2	1.9±0.0
**Res:ChCl 1:1 80% (in water)**	40	2	1.3±0.0
**Res:ChCl 1:1 80% (in water)**	60	2	0.8±0.0

3.2 Yield of SHP and GHP in enzymatic lignin depolymerization at varying lignin and cosolvent concentrations

Table S4: Volumetric and mass related yield of SHP and GHP via enzymatic lignin depolymerization.

Cosolvent	Cosolvent [%] (v/v)	Lignin [g L^{-1}]	SHP+GHP [mg L^{-1}]	SHP+GHP [mg g^{-1}_{Lignin}]
**DMSO**	2	1	21.6	21.6
**DMSO**	5	1	26.5	26.5
**DMSO**	10	1	29.9	29.9
**DMSO**	20	1	32.5	32.5
**DMSO**	30	1	32.2	32.2
**DMSO**	40	1	35.3	35.3
**DMSO**	2	2	34.2	17.1
**DMSO**	5	2	41.1	20.6
**DMSO**	10	2	44.6	22.3
**DMSO**	20	2	52.7	26.4
**DMSO**	30	2	50.0	25.0
**DMSO**	40	2	57.0	28.5
**DMSO**	5	2	32.0	16.0
**DMSO**	10	2	28.7	14.3
**DMSO**	20	2	31.2	15.6
**DMSO**	30	2	31.5	15.8
**DMSO**	40	2	27.8	13.9
**DMSO**	50	2	19.5	9.7
**DMSO**	60	2	0.0	0.0
**DMSO**	5	5	31.1	6.2
**DMSO**	10	5	36.8	7.4
**DMSO**	20	5	51.6	10.3
**DMSO**	30	5	53.6	10.7
**DMSO**	40	5	50.0	10.0
**DMSO**	50	5	32.6	6.5
**DMSO**	60	5	0.0	0.0
**DMSO**	10	10	44.1	4.4
**DMSO**	10	10	36.1	3.6
**DMSO**	20	10	74.7	7.5
**DMSO**	30	10	74.9	7.5
**DMSO**	40	10	88.5	8.9
**DMSO**	50	10	62.2	6.2
**DMSO**	60	10	0.0	0.0
**DMSO**	20	20	59.1	3.0
**DMSO**	20	20	75.6	3.8
**DMSO**	20	20	86.2	4.3
**DMSO**	30	20	119.0	6.0

**DMSO**	40	20	132.1	6.6
**DMSO**	50	20	89.8	4.5
**DMSO**	60	20	0.0	0.0
**DMSO**	30	30	135.7	4.5
**DMSO**	30	30	177.0	5.9
**DMSO**	30	30	164.8	5.5
**DMSO**	30	30	164.9	5.5
**DMSO**	40	30	190.6	6.4
**DMSO**	50	30	115.5	3.9
**DMSO**	60	30	0.0	0.0
**DMSO**	30	10	89.3	8.9
**DMSO**	30	10	80.9	8.1
**DMSO**	30	10	75.2	7.5
**DMSO**	40	40	156.4	3.9
**DMSO**	40	40	118.9	3.0
**DMSO**	50	40	108.4	2.7
**EG:ChCl 3:1**	5	1	9.1	9.1
**EG:ChCl 3:1**	10	1	21.4	21.4
**EG:ChCl 3:1**	20	1	19.1	19.1
**EG:ChCl 3:1**	30	1	21.4	21.4
**EG:ChCl 3:1**	40	1	18.9	18.9
**EG:ChCl 3:1**	50	1	24.7	24.7
**EG:ChCl 3:1**	60	1	16.5	16.5
**EG:ChCl 3:1**	5	2	16.5	8.2
**EG:ChCl 3:1**	10	2	27.4	13.7
**EG:ChCl 3:1**	20	2	35.3	17.7
**EG:ChCl 3:1**	30	2	36.0	18.0
**EG:ChCl 3:1**	40	2	28.4	14.2
**EG:ChCl 3:1**	50	2	33.9	16.9
**EG:ChCl 3:1**	60	2	35.0	17.5
**EG:ChCl 3:1**	10	5	44.7	8.9
**EG:ChCl 3:1**	20	5	49.4	9.9
**EG:ChCl 3:1**	30	5	52.4	10.5
**EG:ChCl 3:1**	40	5	59.7	11.9
**EG:ChCl 3:1**	50	5	61.4	12.3
**EG:ChCl 3:1**	60	5	64.1	12.8
**EG:ChCl 3:1**	20	10	71.1	7.1
**EG:ChCl 3:1**	30	10	63.8	6.4
**EG:ChCl 3:1**	40	10	81.6	8.2
**EG:ChCl 3:1**	50	10	86.2	8.6
**EG:ChCl 3:1**	60	10	90.4	9.0
**EG:ChCl 3:1**	40	20	90.8	4.5
**EG:ChCl 3:1**	50	20	101.5	5.1
**EG:ChCl 3:1**	60	20	86.2	4.3

**EG:ChCl 3:1**	60	30	57.1	1.9
**EG:ChCl 3:1**	60	30	40.2	1.3
**EG:ChCl 3:1**	30	5	58.9	11.8
**EG:ChCl 3:1**	30	5	53.1	10.6
**EG:ChCl 4:1**	5	1	21.5	21.5
**EG:ChCl 4:1**	10	1	23.9	23.9
**EG:ChCl 4:1**	20	1	26.3	26.3
**EG:ChCl 4:1**	30	1	30.2	30.2
**EG:ChCl 4:1**	40	1	32.1	32.1
**EG:ChCl 4:1**	50	1	25.1	25.1
**EG:ChCl 4:1**	60	1	25.0	25.0
**EG:ChCl 4:1**	10	2	35.6	17.8
**EG:ChCl 4:1**	10	2	31.4	15.7
**EG:ChCl 4:1**	20	2	33.2	16.6
**EG:ChCl 4:1**	20	2	38.2	19.1
**EG:ChCl 4:1**	40	2	32.0	16.0
**EG:ChCl 4:1**	40	2	40.6	20.3
**EG:ChCl 4:1**	60	2	29.5	14.8
**EG:ChCl 4:1**	60	2	34.8	17.4
**EG:ChCl 4:1**	10	5	71.6	14.3
**EG:ChCl 4:1**	20	5	74.4	14.9
**EG:ChCl 4:1**	30	5	79.7	15.9
**EG:ChCl 4:1**	40	5	81.1	16.2
**EG:ChCl 4:1**	50	5	72.7	14.5
**EG:ChCl 4:1**	60	5	71.3	14.3
**EG:ChCl 4:1**	20	10	86.1	8.6
**EG:ChCl 4:1**	30	10	89.1	8.9
**EG:ChCl 4:1**	40	10	85.9	8.6
**EG:ChCl 4:1**	50	10	91.3	9.1
**EG:ChCl 4:1**	60	10	82.5	8.3
**EG:ChCl 4:1**	40	20	114.0	5.7
**EG:ChCl 4:1**	50	20	126.4	6.3
**EG:ChCl 4:1**	60	20	111.3	5.6
**EG:ChCl 4:1**	60	30	97.8	3.3
**EG:ChCl 4:1**	30	10	88.6	8.9
**EG:ChCl 4:1**	30	10	80.8	8.1
**EG:ChCl 2:1**	5	1	28.4	28.4
**EG:ChCl 2:1**	10	1	29.0	29.0
**EG:ChCl 2:1**	20	1	27.7	27.7
**EG:ChCl 2:1**	30	1	28.9	28.9
**EG:ChCl 2:1**	40	1	38.1	38.1
**EG:ChCl 2:1**	50	1	35.4	35.4
**EG:ChCl 2:1**	60	1	34.7	34.7
**EG:ChCl 2:1**	5	2	42.8	21.4

**EG:ChCl 2:1**	10	2	34.5	17.2
**EG:ChCl 2:1**	20	2	19.6	9.8
**EG:ChCl 2:1**	30	2	25.5	12.8
**EG:ChCl 2:1**	40	2	24.2	12.1
**EG:ChCl 2:1**	50	2	25.7	12.8
**EG:ChCl 2:1**	60	2	21.4	10.7
**EG:ChCl 2:1**	10	5	37.8	7.6
**EG:ChCl 2:1**	10	5	35.1	7.0
**EG:ChCl 2:1**	20	5	34.2	6.8
**EG:ChCl 2:1**	30	5	38.5	7.7
**EG:ChCl 2:1**	40	5	42.3	8.5
**EG:ChCl 2:1**	50	5	48.5	9.7
**EG:ChCl 2:1**	60	5	46.9	9.4
**EG:ChCl 2:1**	20	10	56.7	5.7
**EG:ChCl 2:1**	20	10	66.3	6.6
**EG:ChCl 2:1**	20	10	49.5	4.9
**EG:ChCl 2:1**	30	10	42.4	4.2
**EG:ChCl 2:1**	40	10	64.6	6.5
**EG:ChCl 2:1**	50	10	64.6	6.5
**EG:ChCl 2:1**	60	10	59.3	5.9
**EG:ChCl 2:1**	40	20	71.7	3.6
**EG:ChCl 2:1**	40	20	67.1	3.4
**EG:ChCl 2:1**	50	20	69.2	3.5
**EG:ChCl 2:1**	60	20	81.9	4.1
**EG:ChCl 2:1**	60	30	100.9	3.4
**EG:ChCl 2:1**	60	30	80.0	2.7
**EG:ChCl 2:1**	60	30	83.5	2.8
**EG:ChCl 2:1**	30	10	98.0	9.8
**EG:ChCl 2:1**	30	10	59.2	5.9
**EG:ChCl 2:1**	30	10	66.3	6.6

3.3 HPLC chromatograms of lignin depolymerization reaction

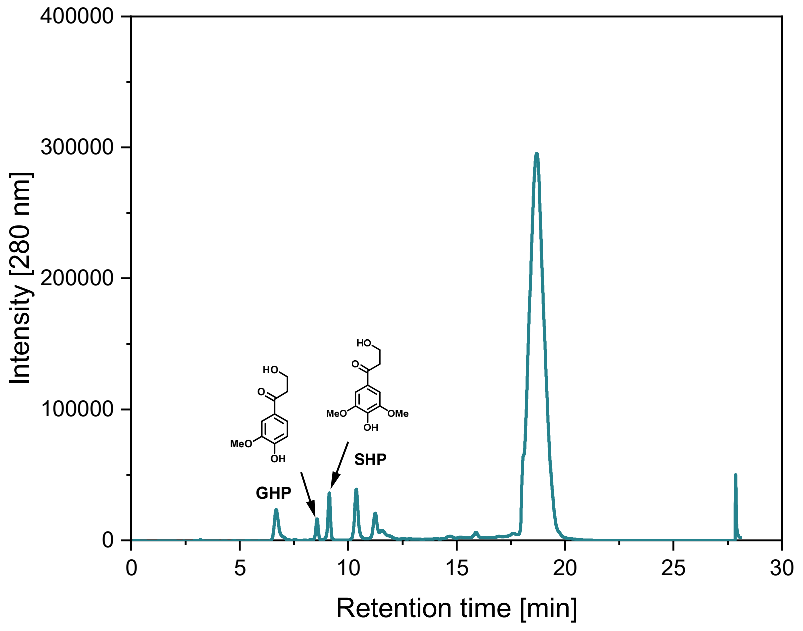

Figure S3: Exemplary HPLC chromatogram of the extract from a lignin depolymerization reaction. The peaks. corresponding to the products GHP (RT = 8.6 min) and SHP (RT = 9.15 min). are highlighted

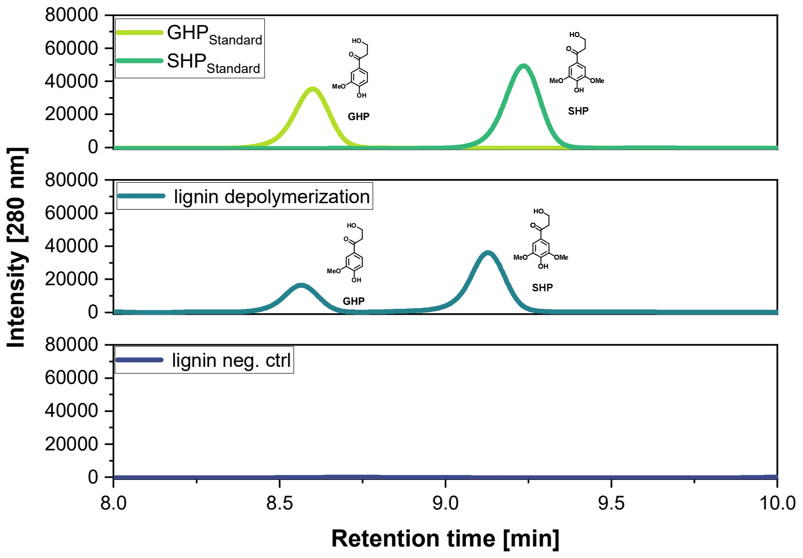

Figure S4: Comparison of chromatograms for GHP (RT = 8.6 min) and SHP (RT = 9.15 min) standards with lignin depolymerization extract and an extract of lignin depolymerization without added enzymes (neg. control). The chromatograms are zoomed in for the critical retention time span for GHP and SHP from 8.0 to 10.0 minutes.

3.4 GC-MS of SHP and GHP from enzymatic lignin depolymerization

To determine the molecular mass of the peaks from the lignin depolymerization, corresponding to GHP (RT = 8.6 min) and SHP (RT = 9.15 min), the peaks were isolated during the HPLC analysis. The isolated peaks were then extracted three times with dichloromethane, and the solvent was evaporated in vacuo. The crude was then dissolved in 1 mL of ethyl acetate and analyzed via GC-MS (Fig. S9, S10).

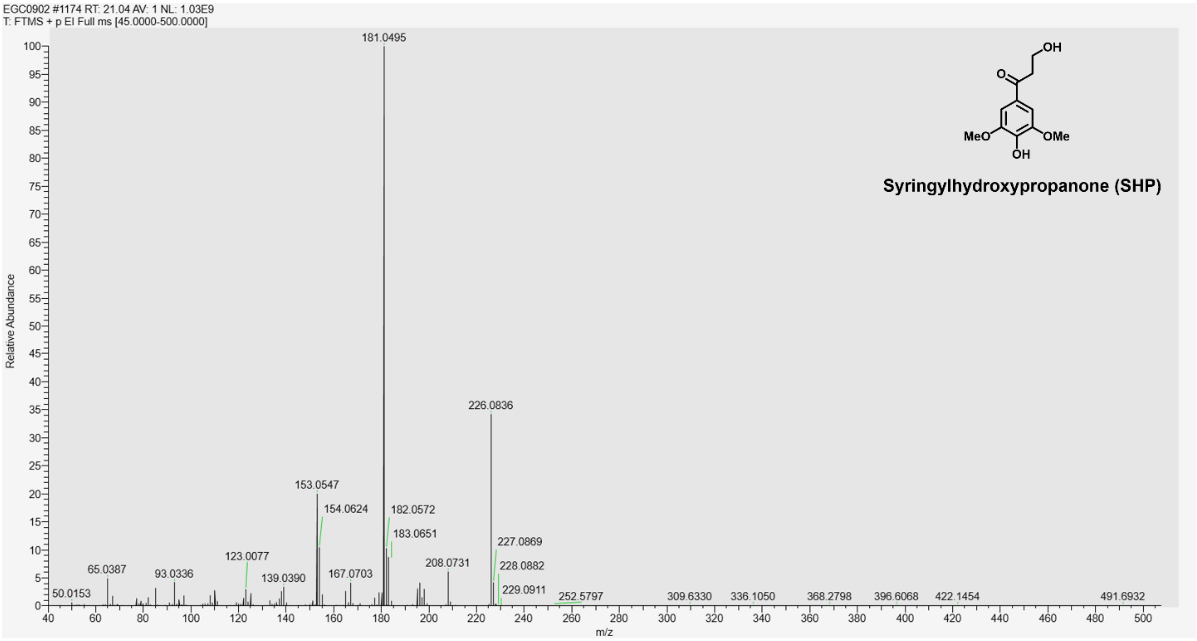

Figure S5: Mass spectrum of the isolated peak from lignin depolymerization with RT = 9.15 min, which corresponds to SHP.

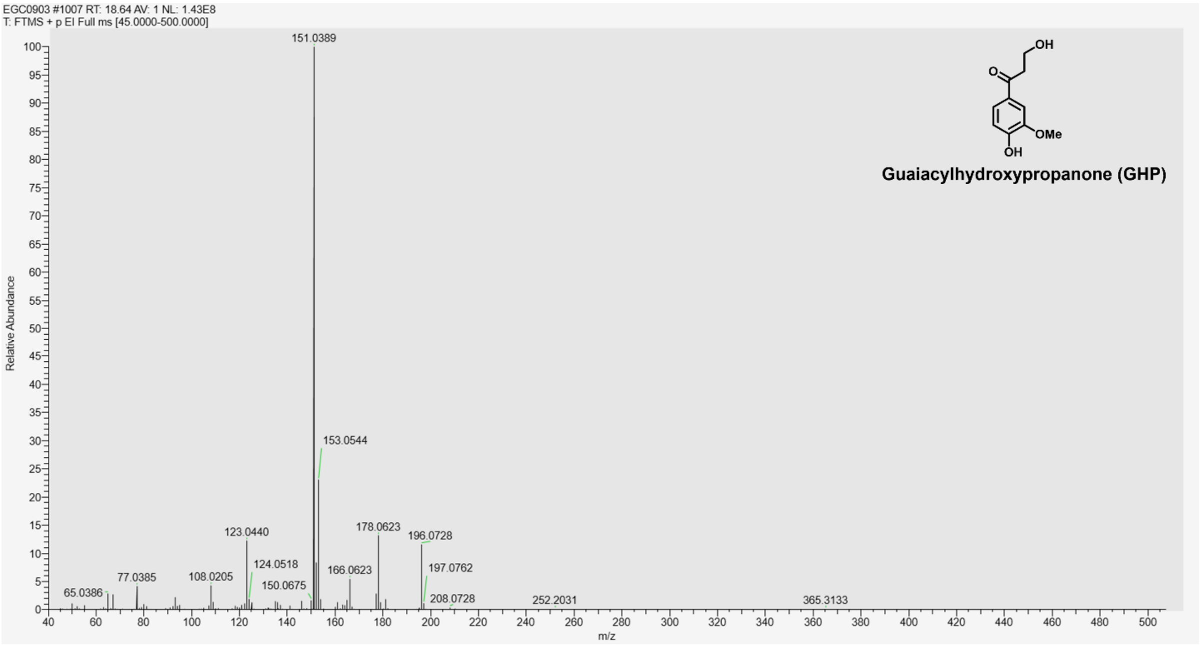

Figure S6: Mass spectrum of the isolated peak from lignin depolymerization with RT = 8.6 min, which corresponds to GHP.

4. Enzyme activity in the presence of DMSO and DES

4.1 Activity of LigD and LigN in the presence of DMSO and DES

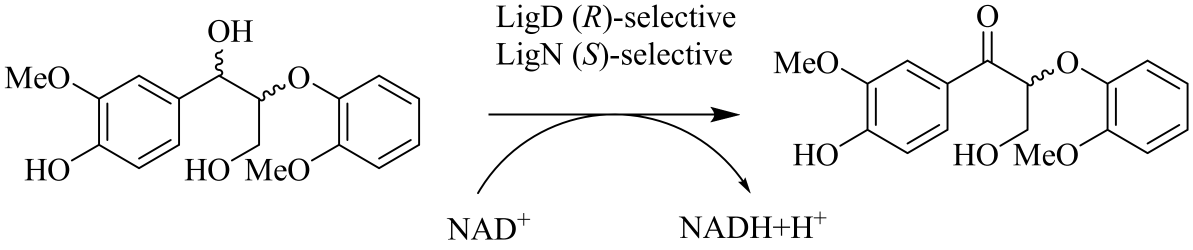

GGE

λ = 370 nm

Figure S7: Oxidation of GGE by the Cα-alcohol dehydrogenases LigD and LigN.

The activity determination of the Cα-alcohol dehydrogenases LigD and LigN was performed in wells of a Microtest Plate 96 Well flat-bottom, obtained from Sarstedt AG (Nümbrecht. Germany). The reaction volume was 200 µL and contained 400 mM glycine/NaOH buffer, pH 9, DMSO or DES in varying volumetric concentrations (1%, 5%, 10%, 20%, 30%, 40%, 50%, 60%), 1 mM GGE (from 100 mM stock solution in DES or DMSO), 4 mM NAD, and 10 µg mL^{-1} of either LigN or LigD. The activity was determined by measuring the absorbance of NADH at 370 nm continuously in a CLARIOstar purchased from BMG Labtech (Ortenberg. Germany) and quantified via calibration curve (Fig. S24).

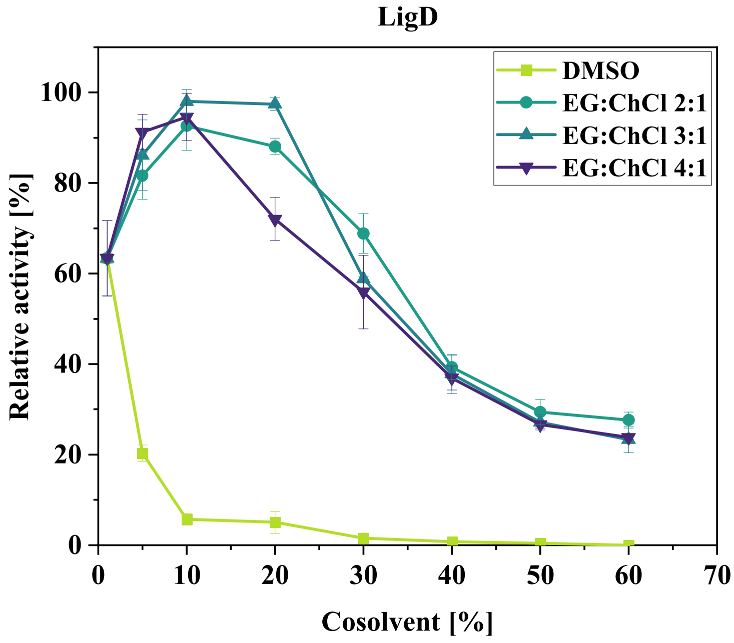

Figure S8: Relative of LigD in the presence of DMSO and EG:ChCl DES in a volumetric concentration range between 1% to 60% (v/v). A relative activity of 100% corresponds to a specific activity of 25.8 U mg^{-1}.

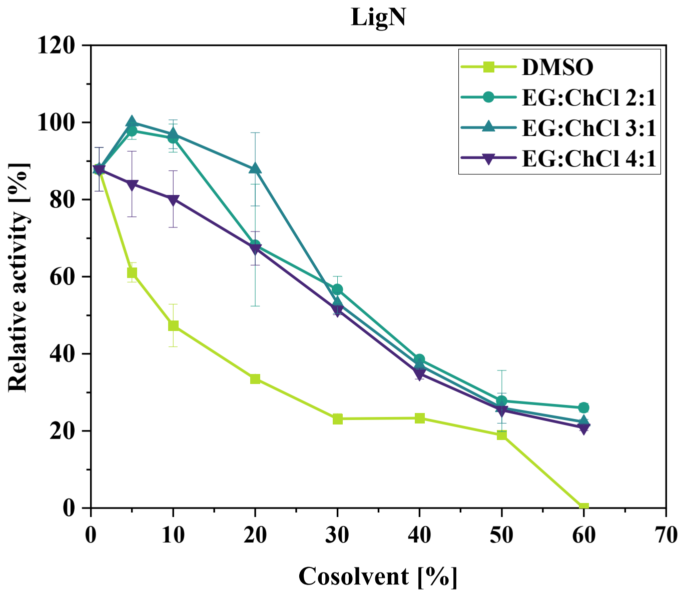

Figure S9: Relative of LigN in the presence of DMSO and EG:ChCl DES in a volumetric concentration range between 1% to 60% (v/v). A relative activity of 100% corresponds to a specific activity of 26.4 U mg^{-1}.

4.2 Activity of LigE-NA and LigF-261 in the presence of DMSO and DES

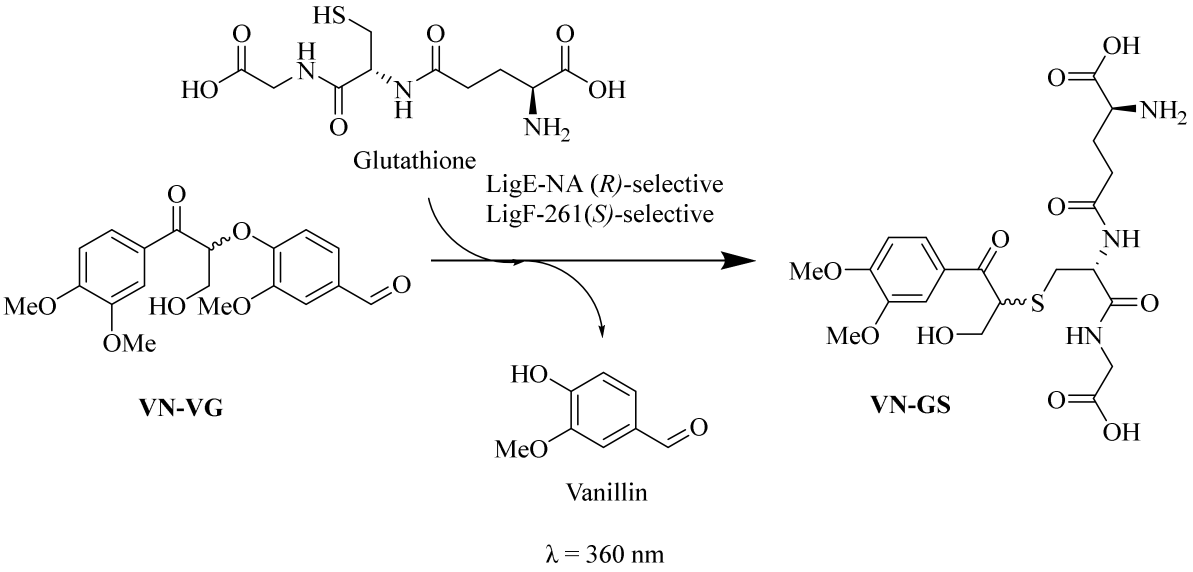

Figure S10: Cleavage of β-O-4 aryl ether linkage in VN-VG by the β-etherases LigE-NA and LigF-261.

The activity of the β-etherases LigE-NA and LigF-261 in the presence of DMSO and the EG:ChCl DES was determined in wells of a Microtest Plate 96 Well flat-bottom, obtained from Sarstedt AG (Nümbrecht, Germany), by measuring the absorbance of released vanillin continuously at 360 nm [2,3]. The reaction volume was 200 µL and contained 400 mM glycine/NaOH buffer, pH 9, DMSO or DES in varying volumetric concentrations (1%, 5%, 10%, 20%, 30%, 40%, 50%, 60%), 1 mM VN-VG (from 100 mM stock solution in DES or DMSO), 1 mM GSH, and 10 µg mL^{-1} of LigF-261 or 20 µg mL^{-1} or LigE-NA. The activity was determined by measuring the absorbance of Vanillin at 360 nm continuously in a CLARIOstar purchased from BMG Labtech (Ortenberg. Germany) and quantified via a calibration curve (Fig. S26).

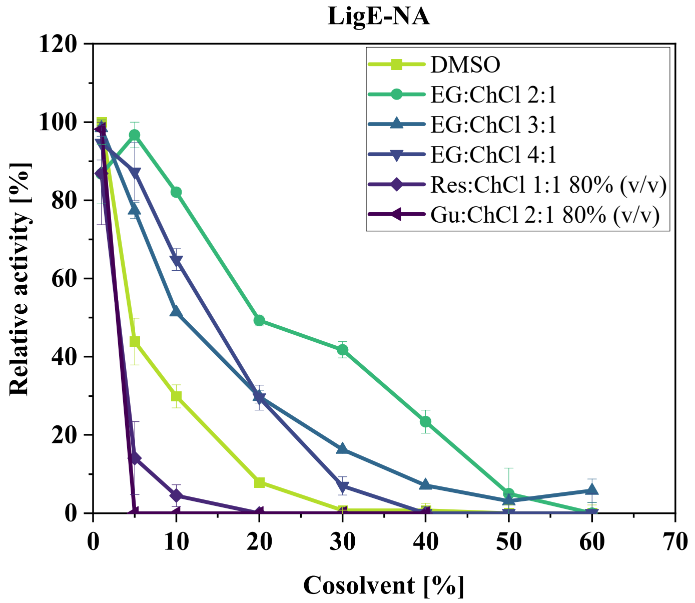

Figure S11*:* Relative of LigE-NA in the presence of DMSO and EG:ChCl DES in a volumetric concentration range between 1% to 60% (v/v). A relative activity of 100% corresponds to a specific activity of 0.93 U mg^{-1}.

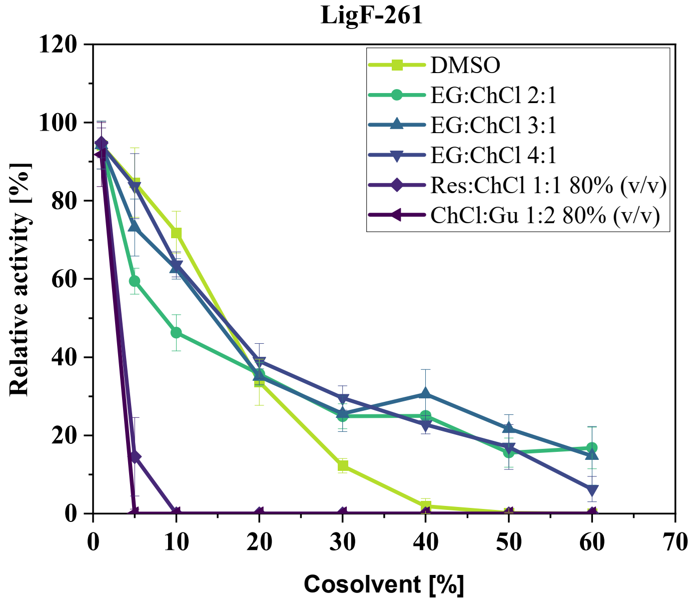

Figure S12: Relative of LigF-261 in the presence of DMSO and EG:ChCl DES in a volumetric concentration range between 1% to 60% (v/v). A relative activity of 100% corresponds to a specific activity of 1.19 U mg^{-1}.

4.3 Activity of TtGAR in the presence of DMSO and DES

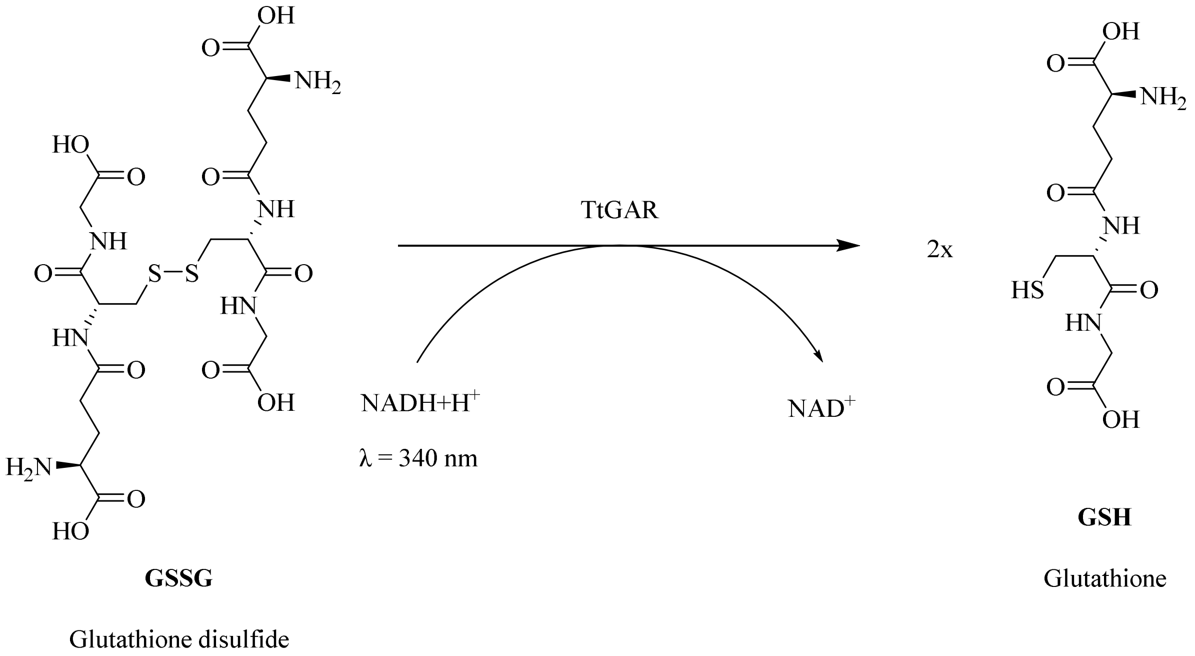

Figure S13: Cofactor recycling by the glutathione amide reductase TtGAR.

The activity of the glutathione amide reductase TtGAR in the presence of DMSO and DES was determined by measuring the decrease in NADH concentration at 340 nm. The assay was performed in 96 Well flat-bottom Microtest Plate wells, obtained from Sarstedt AG (Nümbrecht, Germany). The reactions had a volume of 200 µL and contained 400 mM glycine/NaOH buffer, pH 9, DMSO or DES in varying volumetric concentrations (0%, 5%, 10%, 20%, 30%, 40%, 50%, 60%), 5 mM glutathione disulfide, 0.5 mM NADH, and 100 µg mL^{-1} of TtGAR. The activity was determined by measuring the absorbance of NADH at 340 nm continuously in a CLARIOstar purchased from BMG Labtech (Ortenberg, Germany) and quantified via a calibration curve (Fig. S25).

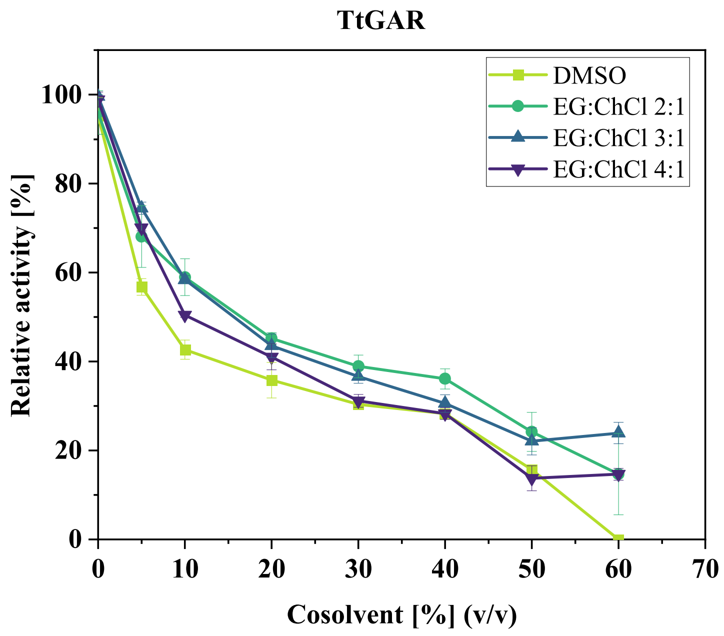

Figure S14: Relative of TtGAR in the presence of DMSO and EG:ChCl DES in a volumetric concentration range between 1% to 60% (v/v). A relative activity of 100% corresponds to a specific activity of 0.04 U mg^{-1}.

4.4 Activity of NaGST$_{Nu}$ in the presence of DMSO and DES

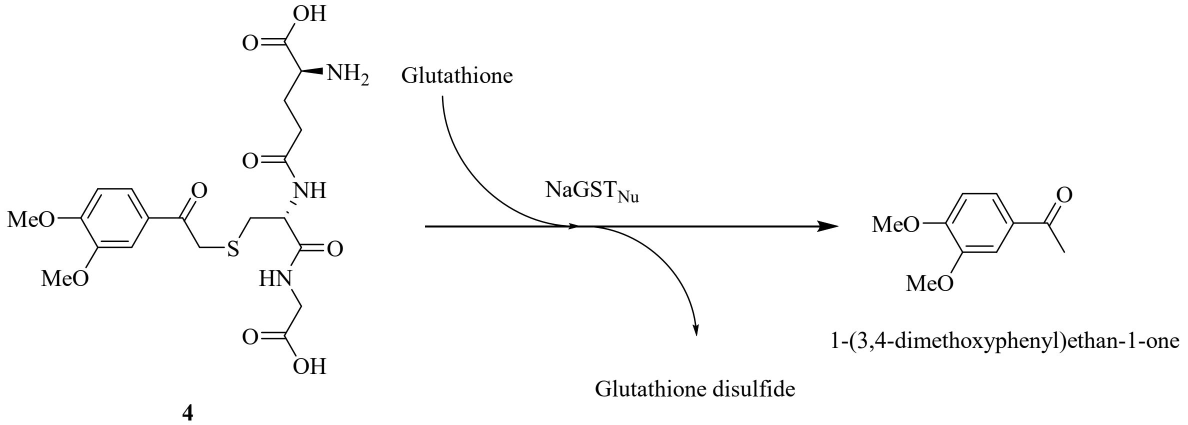

4
GS-βVG
N^5-((*R*)-1-((carboxymethyl)amino)-3-((2-(3,4-dimethoxyphenyl)-2-oxoethyl)thio)-1-oxopropan-2-yl)-*L*-glutamine

Figure S15: Cleavage of the thioether linkage by the glutathione-dependent lyase NaGST$_{Nu}$.

The activity of the enzyme NaGST$_{Nu}$ in the presence of DMSO and DES was determined via the decrease in the substrate concentration GS-βVG (4) via HPLC analysis. The reactions were performed in duplicates in 2 mL Eppendorf reaction tubes with a reaction volume of 1 mL. The reactions contained 400 mM glycine/NaOH buffer, pH 9, DMSO or DES in varying volumetric concentrations (0%, 5%, 10%, 20%, 30%, 40%, 50%, 60%), 1 mM glutathione, 1 mM of the substrate GS-βVG and 2 μg mL^{-1} of NaGST$_{Nu}$. They were carried out at 30°C under shaking at 1000 rpm in a ThermoMixer C from Eppendorf (Hamburg. Germany). The decrease of the substrate's concentration was determined by transferring 50 μL of the reaction mixture every 20 seconds onto 450 μL of a stopping solution (DMSO 250 μL, 2 M HCl). The stopped reactions were then analyzed via reversed-phase high-pressure liquid chromatography on a Nexera XR20 HPLC system from Shimadzu (Duisburg, Germany) using a Nucleosil 100-5 C18 column (250x4 mm) purchased from Macherey Nagel (Düren, Germany). The separation was conducted with an injection volume of 10 μL, a column temperature of 40 °C, a flow rate of 1 mL min^{-1}, and a gradient

elution using a mixture of acetonitrile and TFA-containing water (0.08% (v/v) TFA) as mobile phase. The gradient program was as follows: 0-1 min 10% (v/v) acetonitrile, 1-11 min linear increase from 10% to 100% (v/v) acetonitrile, 11-13 min 100% (v/v) acetonitrile, 13-14 min linear decrease from 100% to 10% (v/v) acetonitrile with an overall measurement time of 14 min. The amount of the substrate was detected using a coupled UV detector at 280 nm and quantified via a calibration curve (Fig. S23).

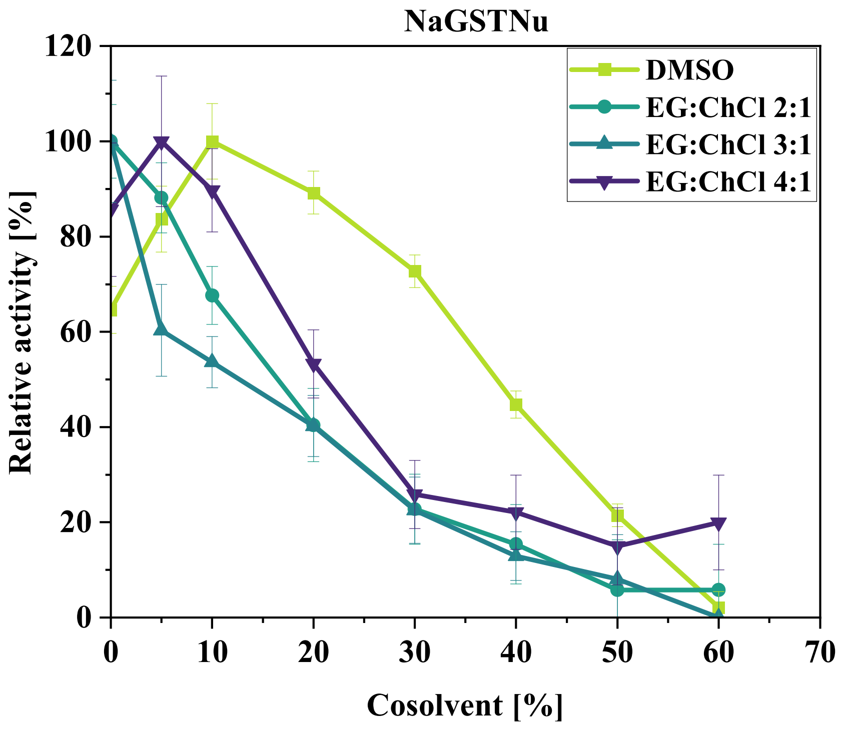

Figure S16: Relative of $NaGST_{Nu}$ in the presence of DMSO and EG:ChCl DES in a volumetric concentration range between 1% to 60% (v/v). A relative activity of 100% corresponds to a specific activity of 200 U mg^{-1}.

5. Synthesis of model compounds

5.1 Chemicals

The compound 2-bromo-1-(3,4-dimethoxyphenyl)ethan-1-one (1) (Fig. S1, S3) was obtained from Angene Chemical (Nanjing, China). Vanillin (Fig. S1) was purchased from Fluorochem (Hadfield, England). Glutathione was obtained from Carl Roth (Karlsruhe, Germany).

5.2 Synthesis of 4-(2-(3,4-dimethoxyphenyl)-2-oxoethoxy)-3-methoxybenzaldehyde

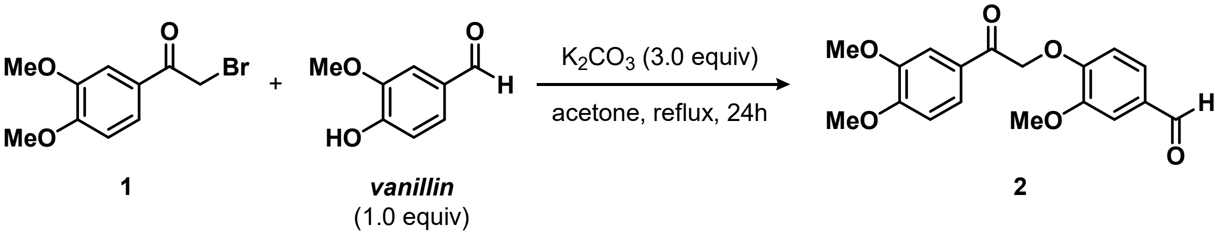

Figure S17: Synthesis of 4-(2-(3,4-dimethoxyphenyl)-2-oxoethoxy)-3-methoxybenzaldehyde.

The Synthesis was adapted from Voß *et al.* (2020) [4] and Gall *et al.* (2014) [2]. To a 250 mL round-bottom flask, 2-bromo-1-(3,4-dimethoxyphenyl)ethan-1-one **1** (5.2 g, 20 mmol) and vanillin (3.0 g, 20 mmol) were added and dissolved in 100 mL of acetone under stirring at room temperature. Potassium carbonate (8.3 g, 60 mmol) was added to the solution, and the reaction was stirred for 24 h under reflux (bath at 60°C). After completion of the reaction, as confirmed by TLC, the solution was filtered to remove excess potassium carbonate, and then the solvent was evaporated *in vacuo*. The dried crude mixture was dissolved in hot ethanol and subsequently cooled down in an ice bath to crystallize the product, yielding *4-(2-(3,4-dimethoxyphenyl)-2-oxoethoxy)-3-methoxybenzaldehyde* **2** (5.3 g, 80% yield) as a white-yellow solid. The product's purity was determined via NMR and compared to that reported by Voß *et al.* (2020) [4] and Gall *et al.* (2014) [2].

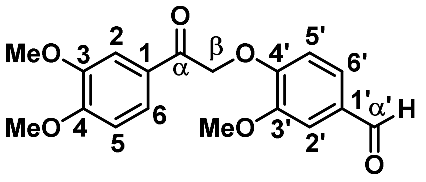

^{1}H NMR (500 MHz, chloroform-*d*) δ 9.85 (s, 1-H, Hα'); 7.66 (dd, 1H, *J* = 10.3, 6.5 Hz, H6); 7.58 (d, 1H, *J* = 1.8 Hz, H2); 7.45 (d, 1H, *J* = 1.7 Hz, H2');

7.38 (dd, 1H, *J* = 9.9, 6.5 Hz, H6'); 6.92 (d, 1H, *J* = 18.5, H5); 6.87 (d, 1H, *J* = 18.5, H5'); 5.42 (s, 2H, Hβ); 3.97 (s, 6H, 4-OMe, 3-OMe); 3.94 (s, 3H, 3'-OMe).

5.3 Synthesis of 4-((1-(3,4-dimethoxyphenyl)-3-hydroxy-1-oxopropan-2-yl)oxy)-3-methoxybenzaldehyde, (VN-VG)

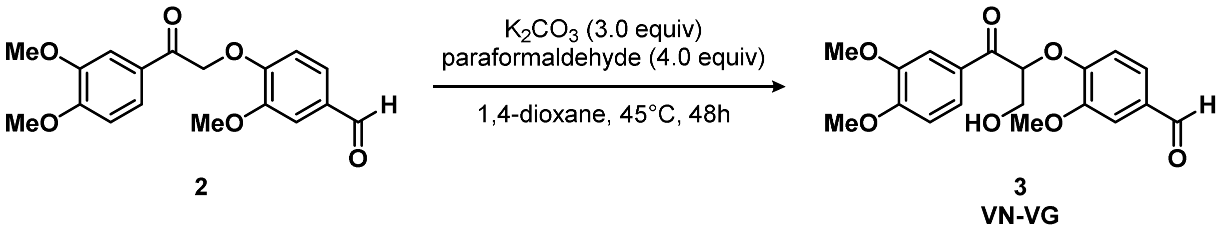

Figure S18: Synthesis of 4-((1-(3,4-dimethoxyphenyl)-3-hydroxy-1-oxopropan-2-yl)oxy)-3-methoxybenzaldehyde (VN-VG).

The synthesis of VN-VG was adapted from Voß et al. (2020) [4] and Gall et al. (2014) [2]. To a 250 mL round-bottom flask, 4-(2-(3,4-dimethoxyphenyl)-2-oxoethoxy)-3-methoxybenzaldehyde **2** (4.95 g, 15 mmol), potassium carbonate (6.2 g, 45 mmol), and paraformaldehyde (1.8 g, 60 mmol) were added. Then 150 mL of dry 1,4-dioxane was added, and the reaction was stirred for 48 h at 45°C. The reaction mixture was filtered to remove the excess of potassium carbonate and paraformaldehyde, and the 1,4-dioxane was then evaporated *in vacuo*. The crude mixture was dissolved in 50 mL of ethyl acetate and washed three times with water and once with brine. The organic layer was dried over anhydrous magnesium sulfate, filtered, and the filtrate was evaporated *in vacuo*. The crude mixture was purified by column chromatography on silica gel using a gradient of pure cyclohexane to cyclohexane/EtOAc (1:1), yielding *4-((1-(3,4-dimethoxyphenyl)-3-hydroxy-1-oxopropan-2-yl)oxy)-3-methoxybenzaldehyde* **VN-VG 3** (3.8 g, 70% yield) as a white solid. The purity of **VN-VG 3** was determined via NMR and compared to that reported by Voß *et al.* (2020) [4] and Gall *et al.* (2014) [2].

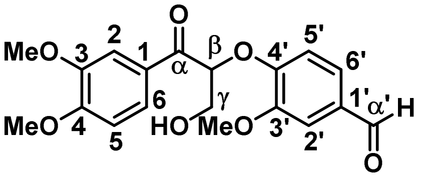

^{1}H NMR (500 MHz, chloroform-*d*) δ 9.86 (s, 1H, Hα'); 8.05 (dd, 1H, *J* = 8.5, 2.0 Hz, H6); 7.70 (d, 1H, *J* = 2.0 Hz, H2); 7.42 (m, 2H, *J* = 8.1 Hz, H2'); 7.39 (d, 1H, *J* = 1.7 Hz, H6'); 6.99 (d, 1H, *J* = 8.1, H5); 6.90 (d, 1H, *J* = 8.61, H5'); 5.42 (s, 2H, Hβ); 3.96 (s, 3H, 4-OMe); 3.92 (s, 3H, 3-OMe); 3.87 (s, 3H, 3'-OMe).

5.4 Synthesis of N5-((R)-1-((carboxymethyl)amino)-3-((2-(3,4-dimethoxyphenyl)-2-oxoethyl)thio)-1-oxopropan-2-yl)-L-glutamine, GS-βVG

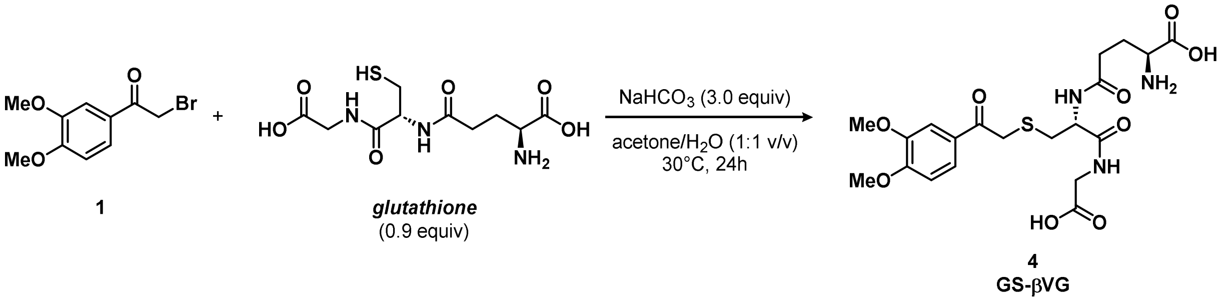

Figure S19: Synthesis of N5-((R)-1-((carboxymethyl)amino)-3-((2-(3,4-dimethoxyphenyl)-2-oxoethyl)thio)-1-oxopropan-2-yl)-L-glutamine.

The synthesis of GS-βVG was adapted from Gall *et al.* (2014) [5]. A 250 mL round-bottom flask was charged with 2-bromo-1-(3,4-dimethoxyphenyl)ethan-1-one **1** (1.7 g, 6.4 mmol) and 30 mL of acetone, and the compound was dissolved under stirring. Then glutathione (1.8 g, 5.8 mmol) and sodium bicarbonate (1.6 g, 19.1 mmol) were added, followed by 30 mL of water. The reaction was stirred for 24 h at 30°C. After the reaction was completed, as confirmed by TLC, 30 mL of dichloromethane was added to the reaction mixture, and the layers were separated using a separatory funnel. The resulting aqueous layer was subsequently extracted three times with dichloromethane. The aqueous layer was freeze-dried, and the resulting crude product was taken up in water. The crude product was then purified using the MPLC Pure C-810 Flash system, purchased from Büchi Labortechnik (Flawil, Switzerland). From the dissolved crude, 5 mL was transferred onto a FlashPure Ecoflex C18 12g obtained from Büchi Labortechnik (Flawil,

Switzerland). A gradient of water and acetonitrile was used for purification, with the concentration of acetonitrile increasing from 10% to 100% over 30 CV at a flow rate of 30 mL min^{-1}. The purification via MPLC was repeated with the same column until the crude was purified entirely. Fractions containing the desired product were unified and subsequently freeze-dried, yielding N5-((*R*)-1-((carboxymethyl)amino)-3-((2-(3,4-dimethoxyphenyl)-2-oxoethyl)thio)-1-oxopropan-2-yl)-L-glutamine **4** (2.2 g, 71% yield) as a yellow-orange powder. The purity of the product was determined via NMR and compared to that reported by Gall et al. (2014) [5].

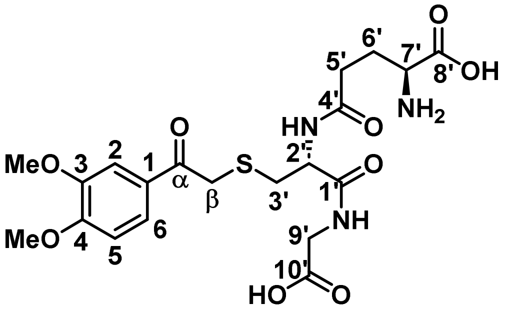

^{1}H NMR (600 MHz, D_2O) δ 7.71 – 7.66 (m, 1H, H6), 7.48 (d, *J* = 2.1 Hz, 1H, H2), 7.08 (d, *J* = 8.5 Hz, 1H, H5), 4.57 (dd, *J* = 9.1, 4.9 Hz, 1H, H2'), 3.94 (s, 3H, 4-OMe), 3.89 (s, 3H, 3-OMe), 3.81 – 3.77 (m, 1H, H9'$_a$), 3.77 – 3.68 (m, 2H, H7', H9'$_b$), 3.59 (dd, *J* = 6.8, 6.0 Hz, 1H, H7'), 3.11 (dd, *J* = 14.3, 4.8 Hz, 1H, H3'$_a$), 2.91 (dd, *J* = 14.2, 9.1 Hz, 1H, H3'$_b$), 2.43 (t, *J* = 7.8 Hz, 2H, H5'$_{a/b}$), 2.07 – 1.97 (m, 2H, H6'$_{a/b}$).

6. Calibration curves

6.1 HPLC calibration curves

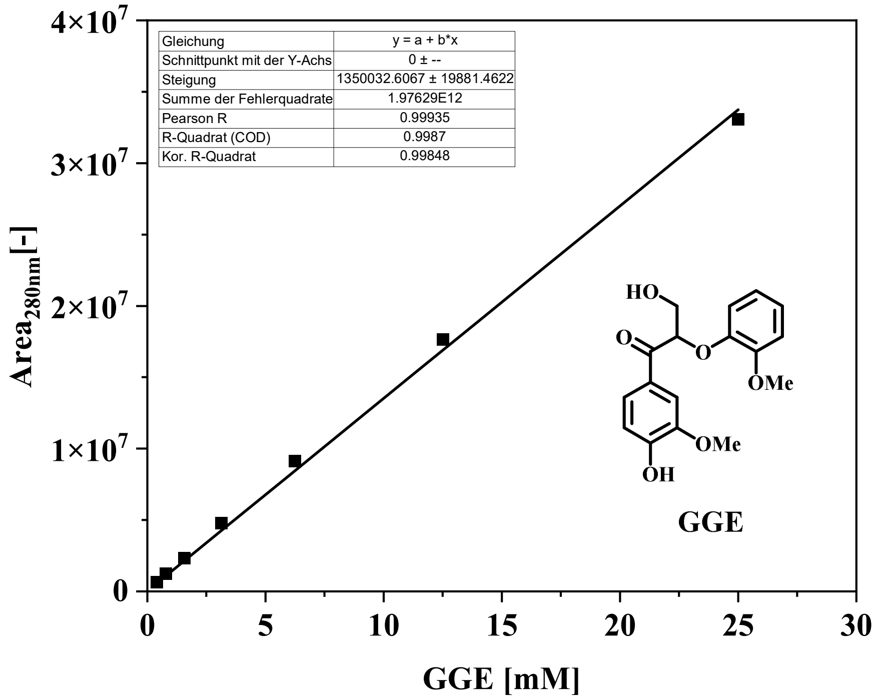

Figure S20: HPLC standard curve for GGE measured at 280 nm.

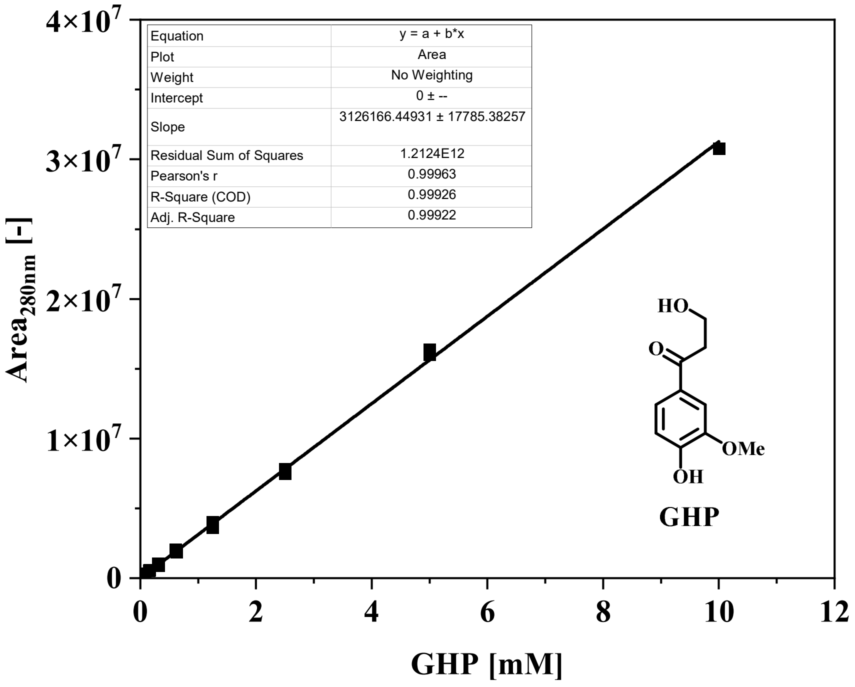

Figure S21: HPLC standard curve for GHP measured at 280 nm.

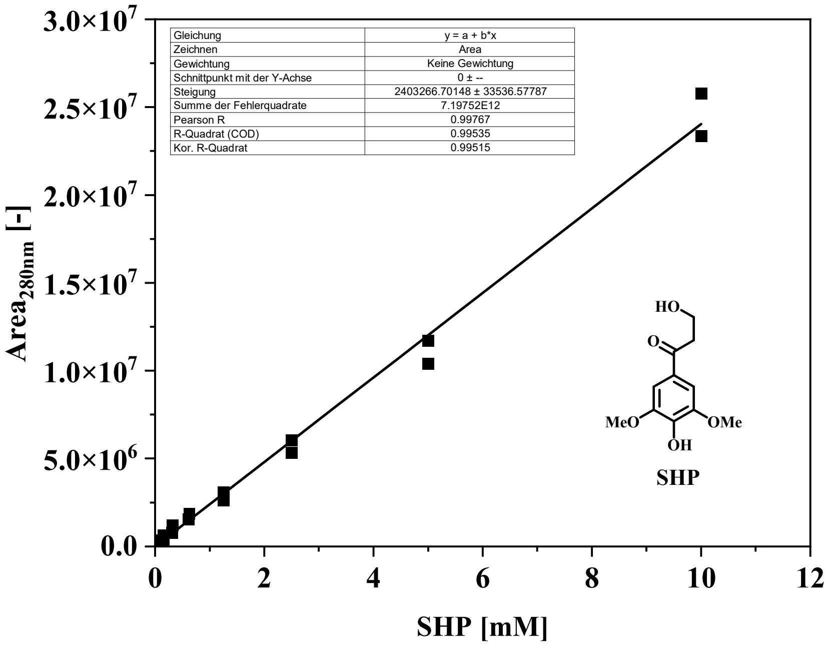

Figure S22: HPLC standard curve for SHP measured at 280 nm.

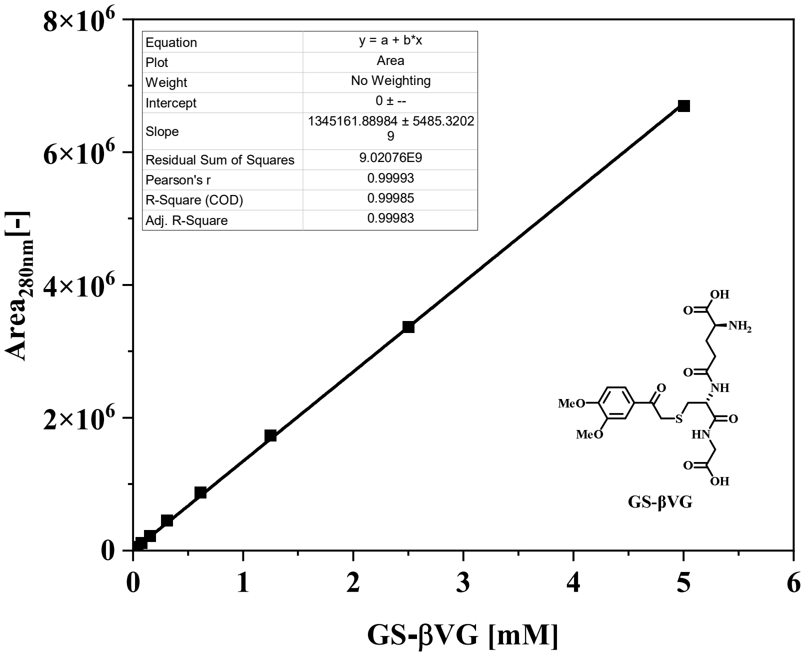

Figure S23: Standard curve for the model substrate GS-βVG, measured at 280 nm via HPLC.

6.2 CLARIOstar calibration curves

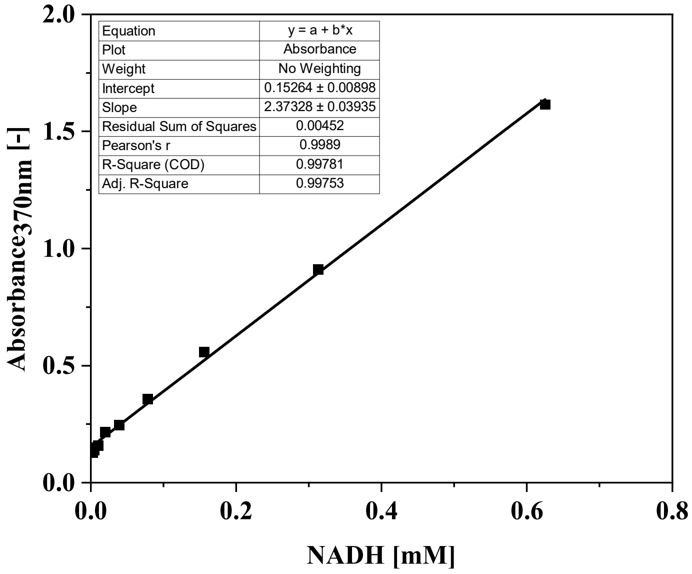

Figure S24: Calibration curve for NADH, measured at 370 nm in a Microtest Plate 96 Well flat-bottom via CLARIOstar.

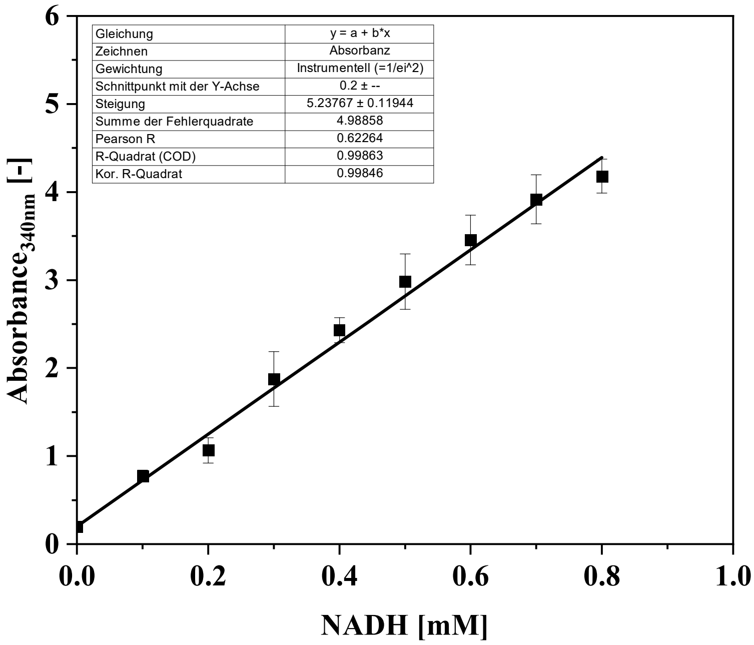

Figure S25: Calibration curve for NADH, measured at 340 nm in a Microtest Plate 96 Well flat-bottom via CLARIOstar.

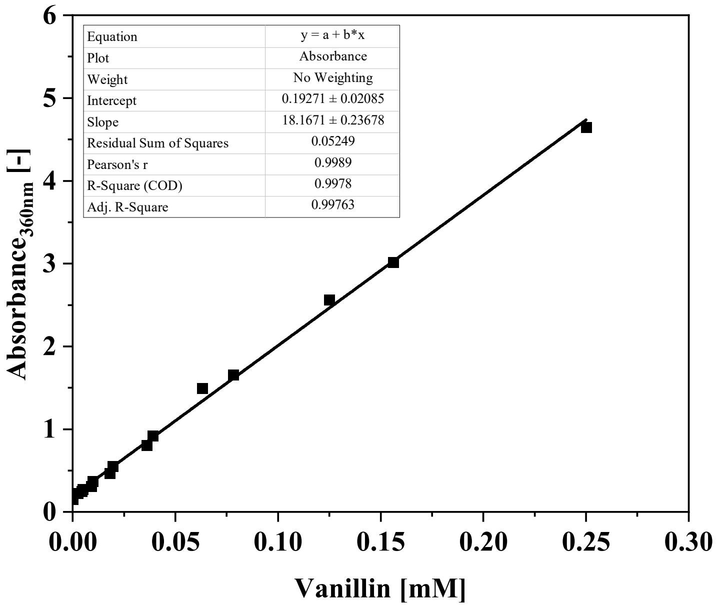

Figure S26: Calibration curve for vanillin, measured at 360 nm in a Microtest Plate 96 Well flat-bottom via CLARIOstar.

7. Molecular dynamics information

7.1 Number of molecules of the MD systems

Table S5: Number of molecules of the MD systems

50% DES	Choline	Chloride	EG	Water	Glycine	c_{HBD}	c_{HBA}
2:1 EG:ChCl	189	189	378	2500	386	0.104	0.052
3:1 EG:ChCl	152	152	456	2500	386	0.125	0.041
4:1 EG:ChCl	128	128	510	2500	386	0.140	0.035
25% DES	Choline	Chloride	EG	Water	Glycine	c_{HBD}	c_{HBA}
2:1 EG:ChCl	95	95	189	2500	386	0.057	0.029
3:1 EG:ChCl	76	76	228	2500	386	0.070	0.024
4:1 EG:ChCl	64	64	255	2500	386	0.078	0.020
12.5% DES	Choline	Chloride	EG	Water	Glycine	c_{HBD}	c_{HBA}
2:1 EG:ChCl	47	47	95	2500	386	0.031	0.015
3:1 EG:ChCl	38	38	114	2500	386	0.037	0.012
4:1 EG:ChCl	32	32	128	2500	386	0.041	0.010

7.2 Code process of KBI calculation

1) Read in RDF for one combination and one run (computed using GROMACS)
2) Application of van der Vegt correction (using pykib python tool)
3) Integration using Kruger correction (adapted from Maginn GitHub)
4) Finding linear regime for extrapolation (using numpy package and own function)
   a) Plot RDF after vdV correction and find distance when RDF becomes flat (using function find_cutoff_radius() that computes gradient of the RDF. From the starting point of r = 0.9 nm the functions evaluates the difference of the gradient to the previous gradient. First limit of extrapolation if the gradient is dg/dr < 0.1 for 7 steps in a row
   b) Using the function find_linear_extrapolation_limit() to identify in G_{ij}-over-(1/r) plot the end of the linear regime: calculating slope at first limit, calculating the gradient along the integral towards 0 and comparing to first slope. Second limit of extrapolation if the difference of the gradient at the first limit and the current is < 0.1 for 7 steps in a row
5) Extrapolation of $G_{ij}(1/r) \rightarrow 0$ using function linregress from scipy.stats (adapted from Maginn GitHub)
6) Loop for 5 repetitions to determine a averaged KIB with standard deviation
7) Repeat calculation for all combination of RDFs

<u>Example: 4:1 EG:ChCl with 50% DES:buffer ratio</u>

The upper figure shows the radial distribution function g(r) over the distance r (nm) between Glycine and Glycine. The five repetitions are shown different colours but here, they lay above each other. The vertical dashed line is the first extrapolation limit and individually set according to step 4 a).

The lower figure plots the Kirkwood-Buff Integral K(r) (nm^3) over the invers distance r^{-1} (nm^{-1}). The dashed line is the same than in the upper figure. The dotted line is the second extrapolation limit and determined according to step 4 b). The extrapolation of $G_{ij}(r^{-1}) \rightarrow 0$ is plotted according to step 5. The average and standard deviation of G_{ij} is given in the legend.

A

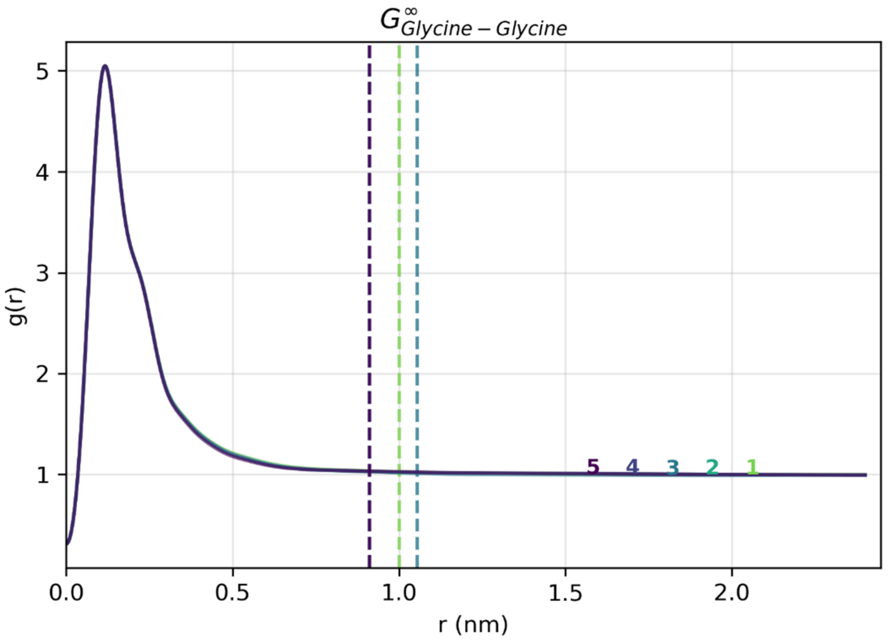

B

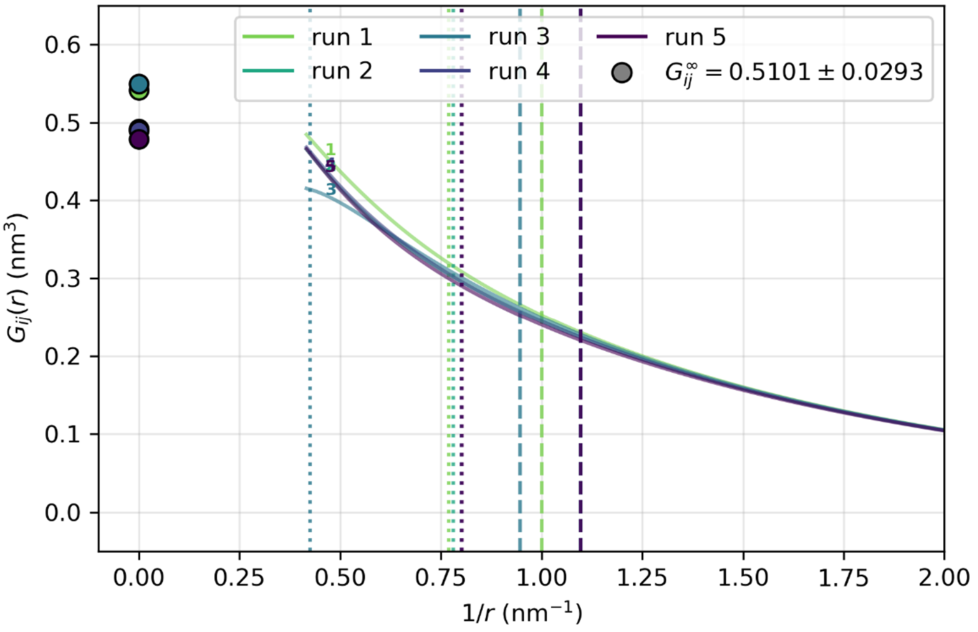

Figure S27: A) Radial distribution function g(r) over the distance r (nm) between Glycine and Glycine for five production runs including first extrapolation limit according to step 4 a). B) Kirkwood-Buff Integral over the inversed distance according to step 4 b) including first and second extrapolation limit of each repetition run.

7.3 Kirkwood-Buff Integral values

Table S6: Values of different Kirkwood-Buff Integrals

	2:1 EG:ChCl. 12.5% DES		2:1 EG:ChCl. 25% DES		2:1 EG:ChCl. 50% DES		4:1 EG:ChCl. 12.5% DES		4:1 EG:ChCl. 25% DES		4:1 EG:ChCl. 50% DES	
Comp.	$\langle G_{ij}^{\infty}\rangle$	$\sigma_{\langle G_{ij}^{\infty}\rangle}$	$\langle G_{ij}^{\infty}\rangle$	$\sigma_{\langle G_{ij}^{\infty}\rangle}$	$\langle G_{ij}^{\infty}\rangle$	$\sigma_{\langle G_{ij}^{\infty}\rangle}$	$\langle G_{ij}^{\infty}\rangle$	$\sigma_{\langle G_{ij}^{\infty}\rangle}$	$\langle G_{ij}^{\infty}\rangle$	$\sigma_{\langle G_{ij}^{\infty}\rangle}$	$\langle G_{ij}^{\infty}\rangle$	$\sigma_{\langle G_{ij}^{\infty}\rangle}$
GGE-EG	2.87	0.57	0.90	0.63	1.94	1.24	0.85	0.36	0.19	0.93	1.56	0.99
GGE-Choline	-4.45	1.17	-1.96	0.38	-0.86	0.48	-0.77	0.51	-2.19	0.18	-3.44	1.21
GGE-Cl	-3.54	1.30	-2.32	0.51	-1.41	0.40	-0.46	1.1	-2.07	0.22	-2.92	1.21
GGE-Gly	1.34	1.15	0.80	0.05	-0.81	1.22	-0.22	0.30	-0.86	0.16	0.93	0.50
GGE-H2O	-0.93	0.46	-1.21	0.07	-0.90	0.25	-0.62	0.11	0.58	0.45	-1.51	0.50
EG-Gly	0.03	0.02	0.05	0.02	0.07	0.02	-0.02	0.03	0.02	0.01	0.01	0.01
Choline-Gly	-0.44	0.02	-0.40	0.02	-0.35	0.03	-0.44	0.08	-0.44	0.02	-0.37	0.03
Cl-Gly	-0.53	0.04	-0.50	0.03	-0.45	0.04	-0.55	0.10	-0.55	0.04	-0.48	0.06
Gly-H2O	-0.16	0.02	-0.18	0.01	-0.21	0.01	-0.13	0.01	-0.18	0.01	-0.18	0.02
Gly-Gly	0.34	0.01	0.43	0.01	0.58	0.06	0.29	0.02	0.40	0.04	0.51	0.03
EG-EG	1.26	0.08	0.72	0.04	0.49	0.01	0.91	0.06	0.5	0.04	0.31	0.03
EG-Choline	-0.39	0.03	-0.33	0.03	-0.29	0.02	-0.37	0.03	-0.37	0.03	-0.30	0.02
EG-Cl	-0.43	0.01	-0.40	0.04	-0.35	0.05	-0.37	0.04	-0.39	0.04	-0.34	0.04
Choline-Choline	1.74	0.10	0.79	0.05	0.38	0.03	2.81	0.34	1.41	0.1	0.72	0.03
Choline-Cl	1.47	0.11	0.84	0.07	0.52	0.05	2.12	0.54	1.37	0.15	0.83	0.04
Cl-Cl	-0.66	0.05	-0.50	0.09	-0.39	0.07	-0.85	0.14	-0.54	0.07	-0.46	0.12
EG-H2O	-0.16	0.01	-0.16	0.01	-0.18	0.01	-0.14	0.02	-0.17	0.01	-0.16	0.01
Choline-H2O	0.08	0.01	0.11	0.01	0.14	0.01	0.07	0.01	0.14	0.02	0.16	0.02
Cl-H2O	0.19	0.01	0.16	0.01	0.17	0.02	0.17	0.01	0.22	0.01	0.20	0.01

References

(1) *The Proteomics Protocols Handbook*; Walker, J. M., Ed.; Humana Press: Totowa, NJ, 2005. https://doi.org/10.1385/1592598900.

(2) Gall, D. L.; Ralph, J.; Donohue, T. J.; Noguera, D. R. A Group of Sequence-Related Sphingomonad Enzymes Catalyzes Cleavage of β-Aryl Ether Linkages in Lignin β-Guaiacyl and β-Syringyl Ether Dimers. *Environ. Sci. Technol.* **2014**, *48* (20), 12454–12463. https://doi.org/10.1021/es503886d.

(3) Seeger, M.; Pagel, J.; Schallmey, A. β-Etherases in Lignin Valorization. In *Methods in Enzymology*; Elsevier, 2025; Vol. 716, pp 239–262. https://doi.org/10.1016/bs.mie.2025.01.050.

(4) Voß, H.; Heck, C. A.; Schallmey, M.; Schallmey, A. Database Mining for Novel Bacterial β-Etherases, Glutathione-Dependent Lignin-Degrading Enzymes. *Appl. Environ. Microbiol.* **2020**, *86* (2), e02026-19. https://doi.org/10.1128/AEM.02026-19.

(5) Gall, D. L.; Kim, H.; Lu, F.; Donohue, T. J.; Noguera, D. R.; Ralph, J. Stereochemical Features of Glutathione-Dependent Enzymes in the Sphingobium Sp. Strain SYK-6 β-Aryl Etherase Pathway. *J. Biol. Chem.* **2014**, *289* (12), 8656–8667. https://doi.org/10.1074/jbc.M113.536250.
